# A structural equation analysis on the relationship between maternal health services utilization and newborn health outcomes: a cross-sectional study in Eastern Uganda

**DOI:** 10.1186/s12884-017-1289-5

**Published:** 2017-03-27

**Authors:** Rornald Muhumuza Kananura, Robert Wamala, Elizabeth Ekirapa-Kiracho, Moses Tetui, Suzanne N. Kiwanuka, Peter Waiswa, Leonard K. Atuhaire

**Affiliations:** 1 Makerere University School of Public Health (MakSPH), Makerere University College of Health Sciences, Kampala, Uganda; 2 Department of Planning and Applied Statistics, Makerere University School of Statistics and Planning, Kampala, Uganda; 3 Epidemiology and Global Health Unit, Department of Public Health and Clinical Medicine, Umeå University, Umeå, Sweden; 4 Global Health Division, Department of Public Health Sciences, Karolinska Institutet, Stockholm, Sweden; 5 Maternal and Newborn Centre of Excellence, Makerere University School of Public Health, Kampala, Uganda

**Keywords:** Interrelationship, Structural equation modeling, Maternal health utilization, Newborn outcomes

## Abstract

**Background:**

Neonatal and maternal health services have a bearing on neonatal mortality. Direct and indirect factors affecting neonatal health outcomes therefore require understanding to enable well-targeted interventions. This study, therefore, assessed the interrelationship between newborn health outcomes and maternal service utilization factors.

**Methods:**

We investigated maternal health utilization factors using health facility delivery and at least four Antenatal Care (ANC) visits; and newborn health outcomes using newborn death and low birth weight (LBW). We used data from a household cross-sectional survey that was conducted in 2015 in Kamuli, Pallisa and Kibuku districts. We interviewed 1946 women who had delivered in the last 12 months. The four interrelated (Endogenous) outcomes were ANC attendance, health facility delivery, newborn death, and LBW. We performed analysis using a structural equation modeling technique.

**Results:**

A history of newborn death (aOR = 12.64, 95% CI 5.31–30.10) and birth of a LBW baby (aOR = 3.51, 95% CI 1.48–8.37) were directly related to increased odds of newborn death. Factors that reduced the odds of LBW as a mediating factor for newborn death were ANC fourth time attendance (aOR = 0.62, 95% CI 0.45–0.85), having post-primary level education (aOR = 0.68, 95% CI 0.46–0.98) compared to none and being gravida three (aOR = 0.49, 95% CI 0.26–0.94) compared to being gravida one. Mother’s age group, 20–24 (aOR = 0.24, 95% CI 0.08–0.75) and 25–29 years (aOR = 0.20, 95% CI 0.05–0.86) compared to 15–19 years was also associated with reduced odds of LBW. Additionally, ANC visits during the first trimester (aOR = 2.04, 95% CI 1.79–2.34), and village health teams (VHTs) visits while pregnant (aOR = 1.14, 95% CI 1.01–1.30) were associated with increased odds of at least four ANC visits, which is a mediating factor for health facility delivery, LBW and newborn death. Surprisingly, newborn death was not significantly different between health facility and community deliveries.

**Conclusions:**

Attending ANC at least four times was a mediating factor for reduced newborn death and low birth weight. Interventions in maternal health and newborn health should focus on factors that increase ANC fourth time attendance and those that reduce LBW especially in resource-limited settings. Targeting women with high-risk pregnancies is also crucial for reducing newborn deaths.

**Electronic supplementary material:**

The online version of this article (doi:10.1186/s12884-017-1289-5) contains supplementary material, which is available to authorized users.

## Background

Globally, at least four million newborns die within four weeks of life every year, of which 75% die within the first week [1]. The largest proportion of neonatal death occurs in low-and-middle-income countries (LMICs) where access to quality health care is low [1, 2]. Most of these newborns die without skilled care that could greatly increase their chances of survival [3]. Access to quality maternal and newborn health services in most of the LMCs is constrained due to various health systems, cultural, socioeconomic, and demographic factors [3]. Though the preventable causes of newborn death such as congenital malformations, tetanus, preterm delivery, diarrhea, pneumonia, intrapartum-related birth asphyxia, and consequences of LBW are well known [1, 4–7], addressing such causes becomes impossible in LMICs where health facilities always experience lack of skilled health workers and stock out of essential medicine and supplies [8, 9].

Uganda’s neonatal death rate is estimated at 27 per 1000 live births, which is higher than that of her neighbors (Kenya at 23 and Tanzania at 21 per 1000 live births) [2]. A wider comparison with other African countries still finds Uganda’s rate high; for example, Egypt has a rate of 11, South Africa, 15 and Morocco 17 deaths per 1000 live births according to a recent UNICEF report on child health outcomes [2]. Low birth weight (LBW) in Uganda is roughly estimated at 11% [10] given that almost a quarter of the newborns are not weighted [11]. LBW has been found to be significantly related to newborn deaths especially in resource-limited settings [4, 11, 12]; yet these could be saved with simple care such as warmth, feeding, hygiene and early treatment of infection [4, 6].

In addition, the utilization of maternal and newborn services such as ANC attendance, skilled delivery assistance, and postnatal care in Uganda is still below average. For example, ANC fourth time attendance, delivery under skilled assistance and postnatal care within 2 days are estimated at 48, 58, and 33% respectively [13]. Yet these services are cornerstones for assessing newborn and maternal danger signs during pregnancy, labor and after delivery [3, 6]. The utilization of these services is challenged by both demand and supply side constraints including; delays to seek care and system inefficiencies such as low staffing levels, poor care skills, frequent supplies and drugs stock outs and the lack of essential equipment [5]. On the demand side, access to finances has been indicated as the most important factor that affects the utilization of maternal and newborn services [14–16]. Studies done in Rwanda and Ghana have indicated the importance of insurance schemes in increasing the likelihood of maternal and newborn skilled service utilization [15, 16]. Women’s access to and control over finances, not only guarantees timely access to services, but also to quality services [15, 16]. In Uganda, it is common for women to deliver at home or within the community because of inhibiting transport costs related to poor community transport systems [17, 18]. Increasing newborn survival, therefore, needs a continuum of care that addresses both the demand and supply side bottlenecks.

The predictors for newborn health outcomes (NHO) and maternal health care utilization (MHCU) are always determined based on approaches that assume direct associations rather than indirect factors [12, 19]. In this study, we considered newborn death (newborn died within 28 days after delivery) and LBW (newborn weight <2.5Kg) for NHOs, while for MHCU, we considered health facility delivery and ANC fourth time attendance. We sought therefore to breach this gap by exploring the interrelationship between factors for newborn outcomes and factors for health facility utilization in rural communities. We believe that health planners, policymakers, and other stakeholders especially from similar settings can use our findings to inform and guide policy formulation needed for the design of interventions aimed at achieving sustainable development goal three. In addition, our findings may contribute to the existing literature particularly on understanding the complexities around newborn death and its relations to low birth weight, health facility delivery, and ANC attendance.

## Methods

### Study design and population

Data for this study was from the end-line cross-sectional survey conducted by Makerere University School of Public Health in July/August 2015 for the evaluation of a Maternal and Neonatal Implementation for Equitable Systems (MANIFEST) project, which was a quasi-exprimental study implemented in the districts of Pallisa, Kibuku and Kamuli in Eastern Uganda from 2013–2015. The project aim was to improve maternal and neonatal health outcomes. The whole of Kibuku district was an intervention area because it only had one health sub-district. Kamuli and Pallisa had three health sub-districts (HSD), and so one HSD was selected as an intervention area and another as a comparison area in each of the district. The district team did the selection of the intervention and comparison area, which was purposively based on the district maternal and new-born service indicators. Data was collected from women who had delivered in the last 12 months irrespective of birth outcomes. The estimated population in the three districts is 1,106,100 [20].

### Sample size and selection of study participants

The study sample size was determined using a two-sided Z-test of the difference between proportions with 80% statistical power, a 5% significance level and 1.5 design effect that resulted in a sample size of 2293 women. The assumption was that after three years (2013–2015) of implementation, skilled deliveries would have increased from 38 to 58%, 62 to 72% and 68 to 78% in the intervention area of Kibuku, Pallisa and Kamuli districts respectively. For the purpose of this study, the data sets for the two project study areas were pooled. However, to verify if the sample size was good enough to measure neonatal death risk factors, we re-estimated the sample size using openEpi [21] at 95% confidence interval with an expected newborn death rate of 27/1000, a precision of 9/1000 (27/1000 ± 9/1000) and a 1.5 design effect which gave us sample size 1867 women. The sample size was, therefore, sufficient to measure newborn death rate and its related predictors.

To select 2293 women, we created a sampling frame of all villages in each of the study areas and estimated 199 villages required to realize the calculated sample size based on an annual average of at least 12 newly delivered women in each village. The 199 villages were selected based on probability proportionate to size. Thereafter, we listed all households in order to identify women who had delivered in the last 12 months irrespective of the birth outcome. During listing, 1946 women were identified as eligible women and all considered for interviews since the estimated sample size was not realized. Trained research assistants made home visits and used a structured questionnaire to interview all eligible women (Additional file 1). The questionnaire was in local language. The principle investigators (RMK, SNK, EEK and MT) and supervisors checked the data collected each day while in the field for completeness. Women whose pregnancies were terminated before 20 weeks were not included. The questionnaires included items for socio-demographic characteristics, antenatal care, socio-economic factors, birth outcome, health system factors, and cultural factors. During analysis, we excluded 52 stillbirths since our focus was on the factors affecting the newborn deaths within 28 days after delivery.

### Study variables

In this study, there were four simultaneous equations having at least one endogenous variable as independent variables in each of the equations. The four interrelated study outcomes (endogenous variables) were newborn death, LBW, at least four ANC visits, and health facility delivery. On the other hand, the exogenous factors were the history of newborn death, receiving VHT home visits, ANC first trimester attendance, gestational age, saving for maternal health, and other socio-demographic characteristics such as religion, occupation, marital status, education, age, gravida, and transport to the health facility.

### Data analysis

We analyzed data using STATA 13.0 in three stages. Firstly, we did a descriptive summary of socio-demographic characteristics, health facility delivery, ANC attendance, newborn death, LBW, and other selected variables. Secondly, we performed a bivariate analysis using *ulogit* STATA command in order to attain the likelihood of the predictors of maternal health utilization and newborn outcomes. Thirdly, we used a generalized structural equation modeling multivariate approach with binomial logit link function since the study had more than one endogenous variable [22]. Only variables that were significant at bivariate analysis and those that were indicated by Marsh et al., 2002 as well as Mosley & Chen frameworks [6, 23] as potential determinants of maternal health utilization and newborn health outcomes were included in the multivariate analysis. We used pairwise correlation matrix to determine the correlation between the independent variables and likelihood ratio test to test for model goodness of fit.

## Results

### Description of study participants

Table 1 presents the description of 1894 mothers of newborns. The majority (92%) of participants were peasants, more than a half (62%) of the respondents had not attained any education and most (91%) were married. In addition, the motorcycle was the most common means of transport used to the health facility (66%) and followed by foot/bicycle (20%). Additionally, 15% were teenage mothers and 14% were of the age 35 and above. Furthermore, 33 per 1000 live births (63/1894) were newborns delivered at the gestational age of 5–7 months and almost two out of five (787/1894) respondents had at least five pregnancies. Fifty-seven per a thousand (109/1894) women had ever experienced a newborn death, 28% (530/1894) had ever received VHT visit after delivery and 35.7% (676/1894) had ever received VHT visit while pregnant. Additionally, 31% (596/1894) accessed ANC in their first trimester and only two percent (46/1894) had saved money for maternal health services.

**Table 1:** Distribution by socio-demographic/economic and health characteristics

Characteristics	*n* = 1,894	Percent (%)
Religion		
Catholic	423	22.3
Muslim	320	16.9
Protestant	823	43.5
Others	328	17.3
Marital status		
Not married	163	8.6
Married	1731	91.4
Education		
None	1177	62.1
Primary	549	29.0
Post-primary	168	8.9
Occupation		
Business	96	5.1
Peasant	1755	92.7
Salaried worker	43	2.3
Age group		
15–19	281	14.8
20–24	634	33.5
25–29	415	21.9
30–34	292	15.4
35–39	194	10.2
40+	78	4.1
Gestational age at birth		
5–6Months	21	1.1
7Months	42	2.2
8Months	271	14.3
9Months	1560	82.4
Gravida		
One	306	16.2
two	296	15.6
Three	284	15.0
Four	221	11.7
Five and above	787	41.6
Type of transport used to the facility		
Motorcycle	1,245	65.7
Vehicle	274	14.5
Others	375	19.8
Had a history of newborn death		
No	1,785	94.2
Yes	109	5.8
Attended ANC Early ^a^		
No	1,298	68.5
Yes	596	31.5
Received VHT visits after delivery		
No	1,364	72.0
Yes	530	28.0
Received VHT visit while pregnant		
No	1,218	64.3
Yes	676	35.7
Saved money for maternal health services		
No	1,848	97.6
Yes	46	2.4
Newborn death^b^		
Alive	1843	97.3
Died	51	2.7
Delivered in the health facility		
No	563	29.7
Yes	1331	70.3
Attended at least 4 ANC visits		
No	724	38.2
Yes	1170	61.8
Low birth weight^c^ *n* = 1442		
No	1249	86.6
Yes	193	13.4

^a^ attended first ANC in first-trimester

^b^ died within 28 days after birth

### Health facility utilization and newborn health outcomes

Seven out of ten (1331/1894) respondents delivered in the health facility and almost four out of ten (724/1894) did not attend all the four ANC visits. Low birth weight was found among 13.4% (193/1442) babies; however, 24% (452/1894) of the newborns were not weighed. Newborn death was estimated to be 27 per 1,000 (51/1894) live births with the majority (83%) dying within seven days after birth. In addition, 33 out of 51 newborn deaths (68%) happened within one day (24 h).

### Maternal health utilization and newborn health predictors at bivariate level

Bivariate analysis was conducted for study variables that had the possibility of being predictors of health facility utilization in the multivariate analysis. Table 2 shows the variable relationships. Factors that significantly increased the odds of health facility delivery were being a salaried worker compared to those that had petty businesses, at least four ANC visits, VHTs visits during pregnancy and saving money for maternal services (*p* < 0.05). The odds of health facility delivery was significantly reduced by being gravida 5+ compared to being gravida one (*p* < 0.05). Regarding the recommended ANC attendance, factors that significantly increased the probability of at least four ANC visits were attendance of ANC in the first trimester, and VHTs visits during pregnancy (*p* < 0.05). Furthermore, factors that significantly reduced the probability of having at least four ANC visits were belonging to the age group of 35+ years compared to the age group 14–19 years (*p* < 0.05).

**Table 2:** Differentials in maternal health utilization by socio-demographic factors and health facility utilization

	Health facility delivery	ANC four-time attendance	Newborn death	Low birth weight
	Unadjusted OR (95%CI)	Unadjusted OR (95%CI)	Unadjusted OR (95% CI)	Unadjusted OR (95% CI)
Religion				
Catholic^a^	1.00	1.00	1.00	1.00
Muslim	0.80 (0.62–1.03)	0.87 (0.68–1.11)	1.44 (0.68–3.05)	1.04 (0.81–1.33)
Protestant	1.04 (0.86–1.27)	1.03 (0.85–1.24)	0.62 (0.32–1.21)	1.11 (0.92–1.34)
Others	1.10 (0.85–1.44)	0.88 (0.69–1.13)	1.86 (0.92–3.76)	0.91 (0.71–1.18)
Marital status				
No married^a^	1.00	1.00	1.00	1.00
Married	0.95 (0.67–1.36)	1.24 (0.90–1.72)	0.65 (0.25–1.69)	0.81 (0.58–1.12)
Education				
None^a^	1.00	1.00	1.00	1.00
Primary	1.25 (0.87–1.80)	1.34 (0.96–1.88)	1.48 (0.57–3.84)	0.63 (0.44–0.90)*
Post primary	1.22 (0.98–1.53)	1.15 (0.94–1.41)	0.34 (0.13–0.88)*	0.75 (0.61–0.93)*
Occupation				
Business^a^	1.00	1.00	1.00	1.00
Peasant	0.88 (0.60–1.30)	0.90 (0.63–1.29)	0.44 (0.18–1.06)	1.27 (0.88–1.85)
Salaried worker	2.65 (1.11–6.33)*	1.82 (0.91–3.64)	2.33 (0.54–9.97)	0.23 (0.09–0.59)*
Age group				
15–19^a^	1.00	1.00	1.00	1.00
20–24	0.94 (0.75–1.17)	1.21 (0.98–1.51)	0.61 (0.27–1.38)	0.98 (0.69–1.39)
25–29	0.91 (0.71–1.15)	1.21 (0.97–1.52)	0.62 (0.26–1.50)	0.40 (0.25–0.64)*
30–34	0.98 (0.74–1.28)	1.01 (0.78–1.31)	0.97 (0.40–2.33)	0.99 (0.65–1.50)
35–39	1.41 (0.99–1.99)	0.69 (0.51–0.93)*	1.26 (0.49–3.25)	1.04 (0.64–1.70)
40+	0.79 (0.49–1.27)	0.61 (0.38–0.95)*	0.59 (0.08–4.36)	1.04 (0.48–2.22)
Had a history of newborn death				
No^a^	1.00	1.00	1.00	1.00
Yes	1.02 (0.67–1.56)	0.81 (0.55–1.19)	12.62 (6.49–24.57)*	1.38 (0.93–2.04)
Attended at least 4 ANC visits				
No^a^	1.00	–	1.00	1.00
Yes	1.42 (1.17–1.75)*	–	0.83 (0.44–1.57)	0.62 (0.45–0.84)*
Attended ANC Early				
No^a^	1.00	1.00	1.00	1.00
Yes	0.95 (0.77–1.17)	3.27 (2.61–4.10)*	0.54 (0.25–1.18)	0.88 (0.64–1.24)
Received VHT visit while pregnant				
No^a^	1.00	1.00	–	1.00
Yes	1.31 (1.06–1.61)*	1.27 (1.05–1.55)*	–	1.03 (0.76–1.42)
Saved for money maternal health services				
No^a^	1.00	1.00	–	–
Yes	1.77 (1.18–2.64)*	1.04 (0.71–1.40)	–	–
Gravida				
One^a^	1.00	1.00	1.00	1.00
Two	0.86 (0.60–1.24)	0.98 (0.70–1.36)	1.15 (0.50–2.62)	1.1 (0.85–1.43)
Three	0.74 (0.51–1.06)	1.03 (0.74–1.44)	0.81 (0.31–2.08)	0.8 (0.61–1.05)
Four	0.84 (0.57–1.25)	1.31 (0.91–1.89)	0.84 (0.30–2.38)	1.06 (0.79–1.42)
Five and above	0.72 (0.53–0.97)*	0.84 (0.64–1.11)	0.75 (0.39–1.45)	1.1 (0.91–1.33)
Gestational age at birth				
5–6Months	–	–	12.01 (3.85–37.47)*	–
7Months^a^	–	–	1.00	–
8Months	–	–	0.66 (0.23–1.87)	–
9Mmonths	–	–	0.43 (0.22–0.85)*	–
Type of Transport used to the health facility				
Motorcycle^a^	–	–	1.00	–
Vehicle	–	–	2.07 (1.09–3.94)*	–
foot/bicycle	–	–	1.25 (0.65–2.42)	–
Low birth weight				
No^a^			1.00	–
Yes			5.56 (2.70–11.44)	–
Delivered in the health facility				
No^a^	–	–	1.00	–
Yes	–	–	1.28 (0.62–2.63)	–
Received VHT visits after delivery				
No^a^	–	–	1.00	–
Yes	–	–	0.45 (0.2–1.07)	–
Baby experienced danger signs				
No^a^	–	–	1.00	
Yes	–	–	0.87 (0.45–1.68)	
Mother experienced danger signs				
No^a^	–	–	1.00	–
Yes	–	–	1.22 (0.65–2.29)	–

OR-Odds Ratio, ^*^ *p* < 0.05, ^a^reference category, dash (−) denotes variables not considered for particular outcome

Regarding newborn health outcomes, factors that significantly reduced the odds of neonatal death were post primary education completion compared to none and gestational age of eight months to nine months compared to the gestational age of 7 months (*p* < 0.05). In addition, factors that increased the odds of newborn death were the history of newborn death, LBW, gestational age at the time of birth of 5–6 months compared to the gestational age of 7 months and use of motor vehicle transport compared to the motorcycle (*p* < 0.05). Factors that significantly reduced the odds of LBW were at least four ANC visits, having at least primary education level compared to none and a salaried occupation compared to petty business.

The predictors of newborn death, LBW, health facility delivery and at least four ANC visits at bivariate level do not reveal causal linkages between the variables. Therefore, a structural equation modeling approach was used to understand the causal linkages as indicated in the next results-sub section. All significant variables identified in the bivariate analysis were considered for further analysis in the multivariate model. However, variables that were not significant such as age group, gravida, education, and occupation were controlled for in the multivariate analysis since available literature has indicated their contribution towards newborn death, low birth weight, and health facility utilization.

### The interrelationship between health facility utilization and newborn health outcomes

Table 3 indicates the details of the interrelationship between health facility utilization and newborn health outcomes. The direct predictors of newborn death were LBW and history of newborn death, while the indirect factors were at least four ANC visits, education, gravida, ANC first attendance in the first trimester of pregnancy, VHTs visits during pregnancy, and religion. Newborns with low birth weight and those born to mothers with a history of newborn death were more likely to die within 28 days after birth (aOR = 3.51, 95% CI 1.48–8.37) and (aOR = 12.64, 95% CI 5.31–30.10) respectively). The odds of the newborn death was not significantly different between those delivered within and outside of the health facilities.

**Table 3:** Analysis of interrelationship between maternal health utilization and newborn health outcomes using GSEM

	Newborn Outcome indicators		Facility utilization indicators	
	Newborn death (Y_1_)	Low birth weight (Y_2_)	Health facility delivery (Y_3_)	ANC 4 time attendance (Y_4_)
	Adjusted OR (95% CI)		Adjusted OR (95% CI)	
Delivered in the health facility				
No^a^	1.00	–	–	–
Yes	0.47 (0.19–1.26)	–	–	–
Attended at least 4 ANC visits				
No^a^	–	1.00	1.00	–
Yes	–	0.62 (0.45–0.85)^**^	1.43 (1.16–1.75)^**^	–
Age group				
15–19^a^		1.00	1.00	1.00
20–24		0.24 (0.08–0.73)^*^	1.04 (0.76–1.42)	1.17 (0.97–1.40)
25–29		0.20 (0.05–0.86)^*^	0.93 (0.66–1.30)	1.20 (0.98–1.47)
30–35		0.34 (0.10–1.17)	1.01 (0.70–1.45)	1.13 (0.91–1.41)
34–59		0.40 (0.11–1.42)	1.27 (0.86–1.86)	0.91 (0.73–1.13)
Attended ANC Early				
No^a^	–	–	–	1.00
Yes	–	–	–	2.04 (1.79–2.34)^**^
Received VHT visit while pregnant				
No^a^	–	–	–	1.00
Yes	–	–	–	1.14 (1.01–1.30)*
Marital status				
Not Married^a^	1.00	1.00	1.00	1.00
Married	0.77 (0.23–2.60)	0.90 (0.53–1.53)	0.94 [0.65,1.36]	1.15 [0.93–1.41]
Education Level				
None^a^	–	1.00	1.00	1.00
Primary	–	0.75 (0.38–1.45)	1.15 (0.76–1.72)	1.18 (0.92–1.50)
Post primary	–	0.68 (0.46–0.98)^*^	1.29 (1.02–1.63)^*^	1.11 (0.97–1.27)
Occupation				
Business^a^	1.00	1.00	1.00	1.00
Peasant	0.54 (0.11–2.65)	0.85 (0.40–1.79)	1.26 (0.80–1.97)	1.14 (0.87–1.49)
Salaried worker	0.42 (0.04–4.60)	1.07 (0.29–3.88)	2.84 (1.03–7.80)^*^	1.50 (0.88–2.53)
Had a history of newborn death				
No^a^	1.00	1.00	–	–
Yes	12.64 (5.31–30.10) ^**^	1.33 (0.72–2.47)	–	–
Gravida				
One^a^	–	1.00	–	–
two	–	0.91 (0.53–1.54)	–	–
Three	–	0.49 (0.26–0.94)^*^	–	–
Four	–	0.65 (0.31–1.37)	–	–
Five and above	–	0.79 (0.39–1.60)	–	–
Type of Transport used to the facility				
Motorcycle^a^	1.00	–	–	–
Vehicle	0.79 (0.20–3.16)	–	–	–
Foot/bicycle	1.40 (0.49–3.93)	–	–	–
Low Birth Weight				
No^a^	1.00	–	–	–
Yes	3.51 (1.48–8.37)^**^	–	–	–
Saved money for maternal health services				
No^a^	–	–	1.00	1.00
Yes	–	–	1.72 (1.14–2.58) ^**^	0.92 (0.74–1.14)

OR-Odds Ratio, ^*^ *p*< 0.05, **p< 0.01, ***p< 0.001, ^a^reference category, dash (−) denotes variables not considered for particular outcome

The factors for LBW as a mediating variable for newborn death were at least four ANC visits, education, gravida, and age. Women who attended ANC at least four times had 38% reduced odds of delivering low birth weight babies compared to those who attended ANC less than four times (aOR = 0.62, 95% CI 0.45–0.85). Mothers who had post–primary education had 32% reduced odds of delivering low birth weight babies compared to those who had no education at all (aOR = 0.68, 95%CI 0.46–0.98). Mothers who were gravida three had 51% reduced odds of delivering low birth weight babies compared to those who were gravida one (aOR = 0.49, 95% CI = 0.26–0.94). LBW was less likely among children born to mothers aged 20–24 (aOR = 0.24, 95% CI 0.07–0.73) and 25–29 (aOR = 0.20, 95% CI 0.05–0.86) years compared to those aged 15–19 years. No significant associations identified among women aged above 29 years.

The factors for attending ANC at least four times as a mediating variable for newborn death and LBW were ANC first attendance in the first trimester of pregnancy, VHTs visits during pregnancy and religion (Table 3). Women who attended ANC in the first trimester were two times more likely to attend ANC at least four times relative to those who attended ANC in the second trimester or later (aOR = 2.04, 95% CI 1.79–2.34). Women who were visited by the VHTs while pregnant had 14% increased odds of attending ANC at least four times (aOR = 1.14, 95% CI 1.01–1.30).

**Fig. 1:**
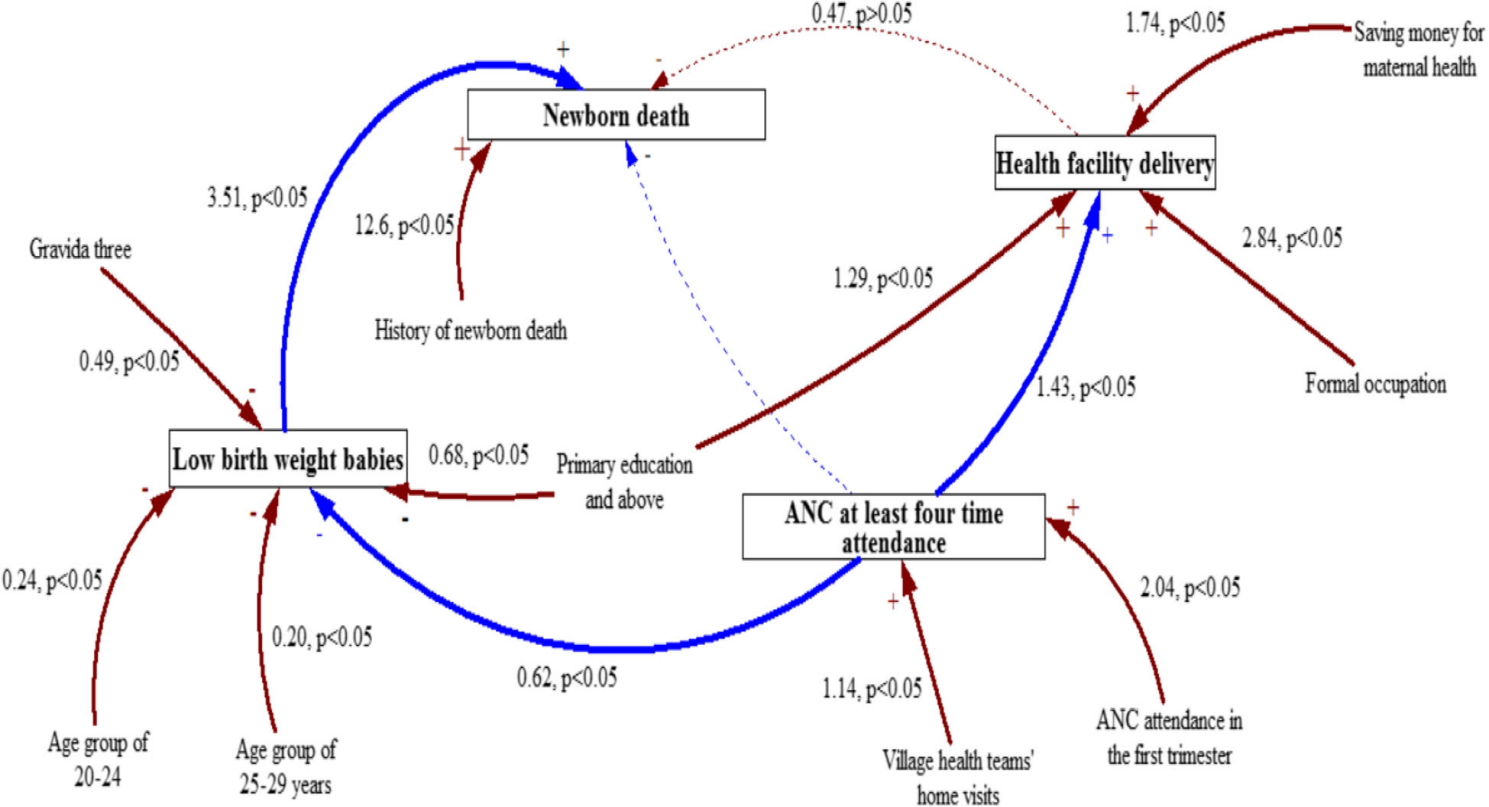
Causal loop for maternal health utilization and newborn health outcome. Note: − negative effect, + positive effect, Negative effect though not significant. Negative effect but dropped because of multicollinearity

Figure 1 is a causal loop diagram that summarizes the interconnectedness between the maternal health care utilization and newborn health outcome factors.

## Discussion

This study has indicated a high proportion of LBW babies in these rural communities and this might be underestimated since almost a quarter of the newborns were not weighed as found in a similar study [11]. Newborn mortality rate was 27 per 1,000 live births, similar to the national average [24]. As noted in similar studies, the newborn mortality rate was high within seven days after delivery (83%) with the majority (68%) of newborns dying within one day [1, 25]. This is an indication of the critical care needed at the time of birth and the immediate period thereafter for newborns to survive [1]. Newborn deaths in the first seven days are mainly attributed to maternal pregnancy and childbirth related complications and infections [12, 26]. Death occurring within one day might be closely related to community and health facility challenges that include delays in seeking care, poor community transport systems, lack of skilled health workers, lack of equipment and drugs at the health facilities and poor referral networks from the lower level to the higher levels [5].

### Interrelationship between newborn outcomes and health utilization factors

The direct determinants of newborn death were LBW, history of newborn death and mother’s age. Since LBW is an endogenous variable, LBW and any other endogenous variable that affect LBW are mediating variables for newborn death. The odds of dying among children born to mothers who had a history of losing newborns was 12 times higher compared to those born by mothers who had no history of newborn death. This was found to be consistent with other studies [24, 27–31]. Other studies have also indicated that women who experienced a pregnancy loss or stillbirth or lost a baby within 28 days in the past are more likely to lose a baby in the subsequent pregnancy [24, 30, 31]. There is, therefore, a need for an examination of historical cohorts of newborns and immediate family members with respect to genetic, chromosomal and congenital predispositions in order to elicit more precise conclusions [32].

The likelihood of increased deaths among LBW newborns was not surprising, since this has been reported in a number of studies done in similar settings [11, 12, 33]. These studies have shown that LBW babies are at a higher risk of a number of health complications, which increase their chances of death if appropriate care is not given. Moreover, the low capacity of health facilities in LMICs to prevent, screen and manage the LBW babies’ complications makes it hard for newborns to survive. Therefore, interventions aimed at building the capacity of health workers on how to screen and care for LBW babies at health facilities are vital.

The factors associated with the odds of LBW as a mediating factor for newborn death were education, at least four ANC visits, mother’s age, and religion. Post-primary level education of mothers was positively related to the birth weight of newborns; a finding that is consistent with existing studies undertaken in other LMICs [34–36]. Lower education levels have been associated with lower health awareness and health seeking behavior as well as low dietary literacy among mothers, which have been found to have a bearing on LBW [35]. Additionally, LBW among babies born to mothers who were gravida three was found to be lower compared to those whose mothers who were gravida one. Similar studies indicated that mothers who are pregnant for the first time are more likely to deliver LBW than mothers with two to four pregnancies [13, 34, 37]. Furthermore, women aged 20–29 years were less likely to deliver LBW babies compared to women aged 15–19 years and there was no significant association established among mothers aged above 29 years. The increased LBW among children born to mothers aged 15–19 years has been associated with the problems faced by such adolescent mothers, who are often women from vulnerable populations, which predisposes them to less access to care [13, 34, 37, 38]. Poor knowledge levels and nutrition status coupled with underdeveloped reproductive organs further increase the chances of adolescents to deliver LBW babies [36]. Additionally, the increased odds of LBW among elderly mothers might be related to poor child spacing [36].

Regarding attending ANC at least four times as a mediating factor for LBW and newborn death, newborns whose mothers attended ANC at least four times were less likely to be LBW babies compared to those who attended for less times, which was consistent with other studies [12, 34, 37]. The World Health Organization (WHO) recommends a minimum of four antenatal visits during pregnancy since this is a period when newborns are affected by problems such as preterm birth, restricted fetal growth and congenital infections that might increase the chances of newborn death [39]. Furthermore, attending ANC has been indicated as a potential avenue for women and their families to receive information and advice on obstetric care as well as identification and management of infections such as Malaria, HIV/AIDS, Syphilis, and other sexually transmitted diseases [39] that have an effect on the fetus. This confirms the importance of conducting population-oriented programs that encourage early ANC attendance [37].

In this study, women who attended ANC in the first trimester had two times the odds of attending ANC at least four times relative to those who attended ANC in the second trimester and above, which was in agreement with a study done in Malawi and Ghana [40]. In addition, the odds of at least four ANC visits was higher among women visited by VHTs while pregnant. This confirms the importance of community health workers in promoting maternal service utilization particularly in rural communities as reported in other studies done in Sub-Saharan Africa [5, 39, 41–45]. These studies have highlighted the importance of community health workers in identifying pregnant women in the community, sensitizing, encouraging and empowering them to seek maternal health services from a qualified health worker. Home visits by VHTs encourages women to attend ANC early (in the first trimester), which helps them benefit fully from preventive strategies, such as iron and folic acid supplementation, treatment of helminthic infections, and intermittent preventive treatment with sulfadoxine-pyrimethamine for malaria in pregnancy among other interventions [39, 46].

Surprisingly, the proportion of newborn deaths for women who delivered in the health facility was not statistically different from those who delivered in the community, which is consistent with recent studies in similar settings [11, 12]. This indifference has been linked to the poor quality of care in the health facilities as well as the possibility of births happening in health facilities but without skilled personnel assistance [33, 47]. This is made worse by system-wide challenges such as drugs and supplies shortages, the lack of essential equipment and shortage of health workers exemplified by a similar study done in Eastern Uganda [48]. This therefore indicates that increasing health facility deliveries without addressing supply side constraints is unlikely to reduce the newborn mortality. In fact, poor quality services at the health facilities reduce utilization of maternal and newborn services. Interventions seeking to improve neonatal health outcomes, therefore, need to consider tackling both demand and supply side constraints.

### Limitations of the study

The analysis was limited to the available information on variables given the nature of the dataset. In addition, the results are generalizable to communities in rural Eastern Uganda but can also be generalized to areas that have a similar context. Additionally, recall bias might have affected the study results but we minimized this by including only women who had delivered in the last 12 months. In addition, excluding 52 records of stillbirth might have had an effect on the health facility and ANC four-time attendance outcomes, however, the records were less than 3% of the total number of participants. The study also did not assess causes of death by timing.

## Conclusions

The interdependence between the predictors of maternal health care utilization and newborn health outcomes highlight important areas of focus for interventions aimed at reducing neonatal mortality. Community health interventions such as home visits by village health teams should be strengthened in order to improve early ANC attendance and recommended ANC visits. There is a need to target women with high risk pregnancies in particular those with a previous history of neonatal mortality and teenage pregnancy. In addition, low cost interventions that improve identification and survival of low birth weight babies in low resource settings should be strengthened.

## Additional file

Additional file 1: Household survey data collection tool. (PDF 930 kb)

## Abbreviations

ANC: Antenatal Care
IRB: Institutional Review Board
LBW: Low birth weight
MakSph: Makerere University School of Public Health
MCHCU: Maternal Health Care Utilization
UDHS: Uganda Demographic Health Survey
UNCST: Uganda National Council for Science and Technology
VHTs: Village Health Teams
WHO: World Health Organization
