# Supplementary material for: A structural equation analysis on the relationship between maternal health services utilization and newborn health outcomes: a cross-sectional study in Eastern Uganda
Source: BMC Pregnancy Childbirth. 2017 Mar 27;17:98. doi: 10.1186/s12884-017-1289-5 (PMC5369185; doi:10.1186/s12884-017-1289-5)
Supplement: Additional file 1: — Household survey data collection tool. (PDF 930 kb) [file 12884_2017_1289_MOESM1_ESM.pdf]

End-term Household Survey July/August 2015-Lusoga Version

	Do you have a woman who gave birth in the last 12 months (including one who delivered a dead child, one whose child died after birth or one whose child is alive) Yes [ ] No [ ] If No, stop and go to the next household otherwise continue with the interview.

		**Questionnaire No.**	[ ]/[ ]/[ ]
		**Household ID No. (as indicated on the listing form)**	[K]/[A]/[ ]/[ ]/ [ ]/[ ]
		**Time Interview Started:**	
		**Time Interview Ededed:**	
	**District**		
	**County**		
	**Sub county**		
	**Parish**		
	**Village**		
	**Name of Head of Household**		

INTERVIEWER VISITS

	**Interview Date**		
	**INTERVIEWER'S NAME**		
	**RESULT***	**1 = COMPLETED 2 = INCOMPLETE INTERVIEW<br>3 = DWELLING NOT FOUND**	
	**NEXT VISIT: DATE**		
	**TIME**		
		**Name**	**Signanture**
	**Checked by:**		

Consent Form

Good morning/afternoon/evening. My name is____________________ (Interviewer)
I am from DHOs office and we are speaking with women and their families about their experiences of being pregnant and of having children. The results of this survey will be used to help improve health programs for women.

Wasuze/osibye otya. Amainha ninze____________ ndikyikilira offisi yomukulu weby'obulamu mu district nga tulikwogeraku nabakyala awalala nabomumaka gaibwe kubumanilivu bwokuba amabundha n'okufuna abana.Ebinava mukunonereza biidha kukozesebwa mukulongosa entegeka dh'ebyobulamu bwabakyala.

You have been selected for the interview by means of a random or chance selection process. I would like to ask you a few questions if I may, but you can refuse to answer any question I ask. You may end the interview at any time. You can also refuse to participate in the study entirely. The interview will last approximately 1 hour. The information we collect from you will be confidential and can only be used for the purpose of the project. **<u>If it is okay with you for us to proceed with the interview, kindly sign below before we start.</u>**

Olondeibwa okubuzibwa ebibuzo nga tukuba akalulu.Nsaba kukubuuza kubibuzo bitono ,aye osobola okuloba okwilamu ekibuzo kyonakyona kyemba nga mbuziza.Osobola okukomya okwilamu ebibuzo esawa yona yona. osobola okuloba okwenhigilamu ilala mukunonereza kunho.okubuzibwa ebibuzo kwidha kutwala sawa nga ndala .Amawulire gonotuwa gaidha kukumibwa nga gakyama era ngaidha kukozesebwa kumugaso kwa pulojekiti eno yonka. Bwewaba nga wazila buzibu nokweyongelayo nebibuzo,nsaba okuntelaku wanho omukono nga tukali kutandika kubibuzo.

May I proceed with the question? Yes ( ) No ( ) . **The respondent should sign as an indication for participating in this study.**
Neyongereyo nebibuzo? Yi ( ) mbee( ) Alikwetaba mukunonenkereza alina okutaku omukono okulaga nti yetabye mukunonenkereza

Signature of the Respondent: ____________________ Signature of Interviewer:_________________

If you have any question about this study, you can contact DHOs office: ________________ Tel: **+256-754-466220**

Instructions: *Every thing in bold or Intalic is instruction for the interviewer. Circle correct answer or write in space provided*

SECTION 1: SOCIAL DEMOGRAPHIC

Qn No.	QUESTION	Fill or circle where applicable		GO TO Q.
101	In what month and year were you born? **Mwezi na mwaka ki gwewazalibwamu?**	MONTH …………../………………		
		Don't know…………..98		
		**YEAR ……../……../……../…………**		
		Don't know…………..98		
102	How old are you? ***Olina emyaka emeka?***	Age ______/_________		
103	What is your religion? ***Osoma idini ki?***	Protestant…………1		
		Catholic……….……2		
		SDA…………….….3		
		Muslims ……….….4		
		Pentecostal/Born again ……….5		
		Others (specify)_________________99		
104	What is your current marital status? ***Oyemereire otya mubyobufumbo buti?***	Single ……………..1		
		Married…………….2		
		Widowed…………..3		
		Divorced…………..4		
		Separated …………5		
105	What is the highest grade of education that you completed? ***Wakoma kwidala ki ely'obwegerese?***	Primary…………….1		
		O'Level …………….2		
		A' level……………..3		
		Tertiary……………..4		
		Vocational…………5		
		None………………..6		
106	What is your primary (main) occupation? *(The occupation where the respondent spends most of her time*) **Mulimoki omukulu gwokola? (Gwosinga okumalaku ebiseera ebisinga)**	Salaried worker ……..1		
		Business……………..2		
		Laborer 3		
		Peasant farmer . . ……4		
		House Wife…………..5		
		Others (specify)……………………99		
107	Do you belong to any community/village saving group/micro finance? ***Olinaku ekibiina kyonakyona ekyokukyalo ekiteleka sente kyolimu?***	Yes …………………1		***If No, go to Question 114***
		No …………………2		

Qn No.	QUESTION	Fill or circle where applicable		GO TO Q.
108	If yes above in Qn 107, what type of saving group is it?<br>***Bwekiba nga yii mukibuuzo 107 waigulu, Kibiina ki?***<br><br>***Circle all mentioned***	Burial group……………………….1<br>Circle group…………..………..2<br>SACCO…………….……………3<br>Microfinance /credit group……….4<br>Farmers' association……………5<br>Mothers/Women saving group……………..6<br>Cooperative society…………….7<br>VSLA……………………..8<br>Others (specify)……………………..99		
109	When did you join the mentioned saving group?<br>***Waingila li ekibina kino ekilikwogerwaku?***	***Type of the saving group (follow the respondent saving groups codes mentioned in 108)***		
		**Saving group type code**	***Month and Year joined***	
		[ ]	Month………..Year………..	
		[ ]	Month………..Year………..	
		[ ]	Month………..Year………..	

Qn No.			
110	How frequently does your saving group collect savings from its members?<br>***Ekibiina kyaimwe kisoloza sente buli luvainuma lwaibangaki?***		
	Type of the saving group (follow the respondent saving groups codes mentioned in 108)	***Frequency codes***<br>*Weekly ....1*<br>*Monthly ....2*<br>*Quarterly 3*	***Amount***
	Saving group type code	**Frequency code**	………………………………
	[ ]	[ ]	………………………………..
	[ ]	[ ]	…………………………………
	[ ]	[ ]	…………………………………
	[ ]	[ ]	
111	*Does your saving group support pregnant women?*<br>***Ekibiina kyaimwe kiyamba abakyala abamabunda?***		
	Type of the saving group (follow the respondent saving groups codes mentioned in 108).	**Codes** *Yes…………1*<br>*No…………..2*	
	Saving group type code	**Response code**	
	[ ]	[ ]	
	[ ]	[ ]	

Qn No.	QUESTION	Fill or circle where applicable		GO TO Q.
112	*If yes, how does your saving group support pregnant women?*<br>***Bwekibanga kibayamba, kiyamba kityaabakyala abali amabunda?***			
	Type of the saving group (follow the respondent saving groups codes mentioned in 108)	***Codes*** *Has MCH account where pregnant women can benefit …………..1*<br>*Has welfare account……………2*<br>*Gives us loans………………..3*		
	Saving group type code	**Response code**		
	[ ]	***[ ]***		
	[ ]	***[ ]***		
113	When did your group start supporting pregnant women? ***Ekibiina kyamwe kyatandiikali okuyamba abakazi abali amabunda?***			
	Type of the saving group (follow the respondent saving groups codes mentioned in 108)	***Year started supporting***	***Amount saved during the saving circle***	
	Saving group type code			
	[ ]	Month……………...Year………………..		
	[ ]	Month……………...Year………………..		
114	How did you save to prepare for your baby's requirements?<br>***Weterekera otya okutegekera ebyetaago ebyomwanawo?***<br>***Circle all mentioned***	Saving group……………..1		
		personal savings(safety box, bank account)……….2		
		Produce/animal……………..3		
		My husband gave me money…………4		
		Others (specify)__________________99		
115	How did you save for transport to the health facility?<br>***Weterekera otya tulansipooti owokukutwala mwidwaliro?***<br>***Circle all mentioned***	Saving group……………..1		
		Individual……………….2		
		Produce/animal……………..3		
		My husband gave me money…………4		
		Others (specify)……………………….99		
116	How did you save for facility bills?<br>***Weterekera otya sente edhokusasula mwidwaliro?***<br>***Circle all mentioned***	Saving group……………..1		
		Individual……………….2		
		Produce/animal……………..3		
		My husband gave me money…………4		
		Expected to get free services from health facility……5		
		Others (specify)……………………99		

Qn No.	QUESTION	Fill or circle where applicable		GO TO Q.
117	Where did you get information on Saving for Maternal and newborn health? **Amawulire agagemaghana kuku tereka sente edh'ebyobulamu mukuzala ni mubana abawere wagatolawa?**	VHTs -through Home Visits……..…1		
		VHTs-through Community Meetings...2		
		Radio………………..3		
		Churches………………..4		
	Circle all mentioned	Health worker/Health facility………5		
		Friend…………………………6		
		Personal knowledhe and Experience…………7		
		Others (specify)………………….……99		
118	Who in your home mainly makes decisions regarding treatment and health care during pregnancy, delivery, and newborn care? ***Ani mumaka munho asalawo kukutereka, obwidandabi nendabirira kubyobulamu mubisera nga oli mabunda, nga oja okuzaala, nokulabirira omwana omughere?***	Mother in-law……………..1		
		Maternal grandmother to baby……….2		
		Husband…………3		
		The mother herself……………4		
	**Circle only one option**	Husband and Wife jointly………..5		
		Others (specify)………………………...99		
119	How did your husband support you while you were pregnant? ***Omwamiwo yakuyamba atya nga olimabunda?***	Gave me money to join saving group…..1		
		Gave me money to buy birth items……………….2		
		Gave me money to buy baby's clothing……………..3		
	Circle all mentioned	Did housework (cooking, washing etc)…………4		
		Bought food……………..-5		
		Escoted her to the health facility…………..6		
		Gave me money transport to the health facility…………7		
		Gave me money to pay facility bills……..8		
		Transported me to the health facility…………9		
		Others (specify)………………………99		

Qn No.	QUESTION	Fill or circle where applicable		GO TO Q.
120	How did your husband support you at the time of delivery? **Omwamiwo yakuyamba atya nga oja okuzaala?**<br><br>***Circle all mentioned***	Escorted/transported me to the health facility…………..1		
		Gave me money transport to the health facility…………2		
		Gave me money to pay facility bills……..3		
		Did housework (cooking, washing etc)…………4		
		Bought food…………………………..5		
		Others (specify)……………………..99		
121	How did your husband get money to support you? **Omwamiwo yafuna atya sente okukuyamba?**<br><br>***Circle all mentioned***	Saving group……………..1		
		Did casual work……………….2		
		Sold our produce/animal/property……..3		
		Saved as in individual…………4		
		Others (specify)………………...……..99		

SECTION 2. KNOWLEDGE

Now I would like to ask you some questions about pregnancy and childbirth. Specifically, I am going to be asking you questions about three different phases that women go through when having a child. These phases are the period of being pregnant, the period of labor and birth, and the period immediately after the birth of the child.
Buti ndigya kukubuza ebibuzo ebigemagana ku mabundha nokuzalibwa kwomwana. Ebibuzo byengya okukubuza bigemagana kumitenedelo esatu omukazi gyabittamu okufuna omwana.Enitendelo nigyinho; ekisera omukazi nga ali mabundha,ekisera ekyokulumwa n'okuzala, n'ekyo nga yakamala okuzala omwana

201	In your opinion, what are the danger signs that can occur during pregnancy that could endanger the life of the woman? **Mundowozayo buzibuki obwebyobulamu obusobola okutuka kumukazi nga alinamabundha nga busobola okukosa obulamu bwe?**<br><br>***Do not read answers. Probe: Anything else?***<br><br>***Circle all mentioned***	Vaginal Bleeding…………….………………..1	***if "don't know-98" go to Question 203***
		Severe headache…………………2	
		Blurred vision …………………….3	
		Convulsions……………..............4	
		Swollen hands/face/feet..………5	
		Fever/Malaria …………...………..….6	
		Unconsciousness……….........7	
		Difficulty breathing ……………..8	
		Severe weakness ……….….……9	
		Severe abdominal pain………..10	
		Accelerated/ reduced Fetal movement……...11	
		High blood pressure……………12	
		Water breaks Without labor ……….13	
		Others (specify)…………………………99	
		Don't Know…………………………98	

Qn No.	QUESTION	Fill or circle where applicable	GO TO Q.
202	From which source(s) did you see, hear, or read about danger signs in pregnancy? **Wabona wa,wawulilira wa,oba wasoma wa ebigemaghana ku bubonero obw'obulabe obubawo nga omukazi alimabundha?**<br><br>**(DO NOT READ THE ANSWERS.) PROBE: Any other sources?**<br><br>**Circle all mentioned**	Radio.................................. 1<br>TV.................................... 2<br>Written Sources e.g posters .... 3<br>Friend/Relative ...... 4<br>Community health worker/VHTs ....5<br>Health Facility /Health worker...........6<br>Community Dialogue meetings………7<br>TBA…………………………..8<br>Others (specify)……………………..99	
203	In your opinion, what are the danger signs that can occur during labor and childbirth that could endanger the life of the woman? **Mundowozayo, buzibuki obwebyobulamu obusobola okutukawo kumukazi ali amabundha ni butta obulamu bwe mukatyabaga nga alikulumwa ni mukuzala ?**<br><br>***Do not read answers. Probe: Anything else?***<br><br>**Circle all mentioned**	Severe bleeding ……………..1<br>Severe headache …………….2<br>Convulsions……………..……..3<br>Fever ……………..……..4<br>Loss of consciousness ……..5<br>Staying in Labor for long…..…...............6<br>Placenta not delivered 30 minutes after baby………7<br>High blood pressure……………8<br>Others (specify)…………………………...99<br><br>Don't Know…………………………98	***if "don't know-98" go to Question 205***
204	From which source(s) did you see, hear, or read about danger signs during labor/child birth? ***Obubaka obwo wabubona, wabughulira,wabusoma gha obugema kububonero obubi mubiseera ebyokulumwa/mukuzaala?***<br><br>**PROBE: Any other sources?**<br><br>**Circle all mentioned**	Radio.................................. 1<br>TV.................................... 2<br>Written Sources e.g posters .... 3<br>Friend/Relative ...... 4<br>Community health worker/VHTs ....5<br>Health Facility /Health worker...........6<br>Community Dialogue meetings………7<br>TBA………………………8<br>Others (specify)…………………...99<br>Don't Know……………..……98	

<table>
<tr><th>Qn No.</th><th>QUESTION</th><th>Fill or circle where applicable</th><th></th><th>GO TO Q.</th></tr>
<tr><td rowspan="14">205</td><td rowspan="14">In your opinion, what are the danger signs that can occur during the first 6 weeks after birth that could endanger the life of the woman? Mundowozayo, buboneroki obwobulabe obuyinza okutukawo munaaku eibili edhisoka nga omukazi yakamala okuzala nga busobola okuta obulamu bwe mukatyabaga?

DO NOT READ THE ANSWERS. Probe: Anything else?

Circle all mentioned</td><td>Severe bleeding ……………….…1</td><td colspan="2" rowspan="14">if "don't know-98" go to Question 207</td></tr>
<tr><td>Severe headache ……..………2</td></tr>
<tr><td>Blurred vision ………..………..3</td></tr>
<tr><td>Convulsions/Amakiro………..……….4</td></tr>
<tr><td>Swollen hands/face………..…5</td></tr>
<tr><td>Fever …………..………6</td></tr>
<tr><td>Bad smelling vaginal discharge …………7</td></tr>
<tr><td>Unconsciousness……………..8</td></tr>
<tr><td>Difficult breathing ……………..9</td></tr>
<tr><td>Severe weakness ……..……10</td></tr>
<tr><td>Severe abdominal pain………..11</td></tr>
<tr><td>Pell/Anemic…………….12</td></tr>
<tr><td>Others (specify)………………….99</td></tr>
<tr><td>Don't Know……………..……98</td></tr>
<tr><td rowspan="11">206</td><td rowspan="11">From which source(s) did you hear, or read about danger signs during the first 6 weeks after birth that could endanger the life of the woman? Obubaka obwo wabubona, wabughulira,wabusoma gha obugema kububonero obubi obukosa obulamu bw'omukyala mubiseera nga omukazi amaze okuzaala?

PROBE: Any other sources?

Circle all mentioned</td><td>Radio.................................. 1</td><td colspan="2" rowspan="11"></td></tr>
<tr><td>TV.................................... 2</td></tr>
<tr><td>Written Sources e.g poster .... 3</td></tr>
<tr><td>Friend/Relative ...... 4</td></tr>
<tr><td>Community health worker/VHTs ....5</td></tr>
<tr><td>Health Facility/Health worker ....6</td></tr>
<tr><td>Community Dialogue meetings………7</td></tr>
<tr><td>TBA…………………………8</td></tr>
<tr><td>Others (specify)………………………99</td></tr>
<tr><td></td></tr>
<tr><td></td></tr>
</table>

Qn No.	QUESTION	Fill or circle where applicable	GO TO Q.
	**Now, I would like to ask you a few questions about the health of newborn babies**. *Nsaba kukubuza ebibuzo bitono ebigemagana kubyobulamu bwabana abawere*		
**207**	In your opinion, what are the danger signs that can occur in a newborn baby from birth up to one month that could endanger the life of a newborn baby? **Mundowozayo, buboneroki obwobulabe obuyinza okutukawo kumwana omuwere okuva lw'azaliibwa paka kumwezi nga busobola okuta obulamu bwe mukatyabaga?**<br><br>***DO NOT REAS THE ANSWERS. Probe: Anything else?***<br><br>**Circle all mentioned**	Difficult or fast breathing………1	***If don't know, then SKIP to 209***
		Yellow skin/eye color (jaundice) ………………..2	
		Poor sucking or feeding ……….3	
		Pus, bleeding, or discharge from around the umbilical cord……………………….4	
		Baby very small ………………..5	
		Skin lesions or blisters……..….6	
		Convulsions………..7	
		Rigidity (stiff)/spasms………………8	
		Lethargy (weak/floppy)) ………………9	
		Unconsciousness ……………10	
		Red or swollen eyes with pus……11	
		Fever…………………….12	
		Feeling hot/cold………………13	
		Anemic/pale…………………14	
		Others (specify)…………………………..99	
		Don't Know…………………..98	
**208**	From which source(s) did you hear, or read about danger signs after birth that could endager the life of a newborn? **Wabona wa,wawulilira wa,oba wasoma wa ebigemagana ku bulabe obusobola okutta obulamu bw'omwana omughere mukatyabaga?**<br><br>**DO NOT READ THE ANSWERS**. ***PROBE: Any other sources?***<br><br>**Circle all mentioned**	CHWs-through Home Visits..1	
		Community Health Workers-through Community Meetings……2	
		Radio………………..3	
		Churches/mosques………………..4	
		Health worker/Health Facility…………… 5	
		Friend………………………….6	
		TBA………………………….7	
		Others (specify)…………………….99	

Qn No.	QUESTION	Fill or circle where applicable	GO TO Q.
	**Knowledge on newborn care practices**		
**209**	In your opinion, what are some of the things a woman can do to prepare for birth? ***Mundowoozayo, bintuki ebimu kumukazi byalinokukola okwetegekera okuzaala?***	Identify mode of transport……1	***If don't know, go to Qn 211***
	DO NOT REAT THE ANSWERS: Probe: Anything else?	Save money ………………2	
		Buying Item for a baby ……..3	
		Identify skilled provider/Place of birth…..4	
		Others (specify)…………………….. 99	
	**Circle all mentiond**	Don't Know………………….98	
**210**	From which source(s) did you see, hear, or read about birth preparedness? ***Wabona wa,wawulilira wa,oba wasoma wa kubintu ebigemagana ku kwetegekera okuzaala?***	Radio.................................. 1	
		TV.................................... 2	
		Written Sources e.g posters 3	
		Friend/Relative 4	
		Community health worker/VHTs 5	
	**DO NOT READ THE ANSWERS. CIRCLE ALL MENTIONED**	Health Facility/ Health workers...........6	
		Community Dialogue meetings………7	
		TBA……..8	
		Others (specify)……………………………..99	
**211**	How long after birth is the baby supposed to start breast feeding? ***Omwana yetaga okutandika okumuta kwibeere nga ghabisewo kiseraki nga omaze okuzaala?***	Immediately (less than 1 hour)…………1	
		Within 1- 6 Hours………..2	
		With 7 – 24 Hours……..3	
		More than 24 Hours ……….4	
**212 (a)**	What should be used for cleaning the baby's cord immediately after birth? ***Kiki ekirinokukozesebwa okuyonda omudondo gw'omwana nga amaze okuzalibwa?***	Nothing…………………………………1	
	PROBE: What else?	Medical drugs……………………..2	
		Powder…………………………3	
	**Circle all mentioned**	Ash………………………………4	
		Salty Water………………………..5	
		Surgical spirit……………………6	
		Water with soap…………….7	
		Water…………………..8	
		Others (specify)…………………..99	

Qn No.	QUESTION	Fill or circle where applicable	GO TO Q.
212 (b)	Should anything be put on the baby's cord to help it heal? ***Eriyo ekirinokutebwa kumudondo gwomwana okuguyamba okuwona?***	Yes………….1	
		No……………2	
213	When should the baby be bathed after birth? **Omwana alinakunazibwa nga ghabise ibangaki nga yakamala okuzaalibwa?**	Immediately (Less than 1 hour)………….1	
		Within 1- 6 Hours…………………………..2	
		Within 7 -24 Hours…………………………..3	
		More than 24 Hours ……………… 4	
214	From which source(s) did you see, hear, or read about newborn care practices? **Wabona wa,wawulilira wa,oba wasoma wa kububonero obubi ebigemaghana ku by'amabundaokuzala?**<br><br>**PROBE: Any other sources?**<br><br>**Circle all mentioned**	Radio.................................... 1	
		TV...................................... 2	
		Written Sources e.g Posters 3	
		Friend/Relative 4	
		Community health worker/VHTs 5	
		Health Facility/ Health workers 6	
		Community Dialogue meetings………7	
		Others (specify)……………………..99	

Setion 3: Births and Newborn Deaths

Now I would like to ask you some more questions about the pregnancies you had in your life time and in the last 12 months, focusing on pregnancies that resulted in babies born alive, babies born dead or the Miscarriages you got .
Buti nsaba kukubuza kubibuzo ebindi ebigemagana kundha dhobaire nadho mubulamubwo bwonabwona mu mwezi eikumi nebili egyibise,nga esila ndhita kubana abazalibwa nga balamu nabo abazalibwa nga baffu.

Previous birth history

301(a)	*Number of pregnancies in the life time of the mother?* ***Omughendo ogwamabunda omukazi geyakafuna mubulamu bwe?***	………………………………………	
301(b)	How many of these pregnancies were ***Mabunda gamirundi emeka kugano aga***		
	Carried up to at least 36 weeks	………………….	
	Carried up to 28-36 weeks	………………….	
	Below 28 weeks	………………….	
301 (C)	How many were born alive or dead? ***Bameka kubo abazaalibwa nga balamu oba nga bafu***		
	Born alive	………………….	
	Born dead	………………….	

Qn No.	QUESTION	Fill or circle where applicable		GO TO Q.
302	Of those born alive and died later, when did they die? *Abo abazalibwa nga balamu nibafa oluvainum, bafali?*			
	Died with 28 days after birth	…………………		
	Died after 28 days after birth	…………………		
	**Now we are going to talk about experiences related to the delivery you had in the last 12 months** ***Buti tuja kwogeraku kubigema kubyemubiseemu mubyokuzaala mumwezi 12 egibise***			
303	How old was the pregnancy at the time of delivery *(we are considering the most recent delivery*)? ***Wewazalira endha yali yamyezi emeka(Tulikutegeeza oluzaalo olusembayo)?***	**[ ]/[ ]** Months		
304	Birth type	Singleton……………….1	***If Singleton, Answer Qn 305 to 310***	
		Multiple………………2	***If Multiple, Answer Qn 311 to 317***	
	**Single births**			
305	Ask for date of birth ***Enaku edh'omwezi***	Day________/Month______/Year_______		
306	Baby's Sex **Ekikula eky'omwana**	Male…………………..1		
		Female…………………2		
307	Was baby born alive or Dead? **Omwana yazalibwa nga mulamu oba nga mufu?**	Dead……………1	**If Dead, Go to section 4**	
		Alive……………2		
308	If the baby was born a live, Is (NAME) still live? **Omwana bwaba yazaalibwa nga mulamu, akali mulamu?**	Yes…………….1	**If Yes, Go to section 4**	
		No…………..2		
309	if the baby is not alive, date of Death	Day………../Month………../Year………		
310	After how long after birth did the baby die ? ***Omwana yafa nga ghabisewo ibangaki nga amaze okuzaalibwa?***	*Within 6 Hours?..................1*		
		Within 7 hours-24hour?................2		
		Within 24hours-7 days?................3		
		Within 7 days to 28 days…………..4		
		After 28 days ………..5		
311	Place of Death ***Ekifo kyeyafiramu***	Gvt Hospital………………..1		
		Gvt Health Centre……………2		
		Mission Hospital………….3		
		Mission Health centre………..4		
		Private Hospital………….6		
		Private clinic…………7		
		Home…………………8		

Qn No.	QUESTION	Fill or circle where applicable	GO TO Q.
		Along the way..........9	
		TBA....................10	
	**Multiple Births**		
312	Ask for date of birth ***Enaku edh'omwezi***		
	a) First birth	Day________/Month______/Year_______	
	b) Second birth	Day________/Month______/Year_______	
313	Baby's Sex ***Ekikula eky'omwana***		
	a) First birth	Male............1 Female...........2	
	b) Second birth	Male............1 Female...........2	
314	Was baby born alive or Dead? **Omwana yazalibwa nga mulamu oba nga mufu?**		
	a) First birth	Dead.........1 Alive.........2	
	b) Second birth	Dead.........1 Alive.........2	
315	If the baby was born a live, Is (NAME) still live? **Omwana bwaba yazaalibwa nga mulamu, akali mulamu?**		
	a) First birth	Yes............1 No.............2	**Only applicable if 313 b) is 2**
	b) Second birth	Yes............1 No.............2	**Only applicable if 313 a) is 2**
316	if the baby is not alive, date of Death ***Omwana bwaba nga timulamu enaku dheyafiraku***		
	a) First birth	Day.........../Month.........../Year..............	**Only applicable if 314 a) is 2**
	b) Second birth	Day.........../Month.........../Year..............	**Only applicable if 314 b) is 2**
317	After how long after birth did the baby die ? ***Omwana yafa nga ghabisewo ibangaki nga amaze okuzaalibwa?***		
	**a) First birth**	*Within 6 Hours?..................1*	**Only applicable if 314 a) is 2**
		Within 7 hours-24hour?................2	
		Within 24hours-7 days?................3	
		Within 7 days to 28 days.............4	
		After 28 days 5	
	**b) Second birth**	*Within 6 Hours?..................1*	**Only applicable if 314 b) is 2**
		Within 7 hours-24hour?................2	
		Within 24hours-7 days?................3	
		Within 7 days to 28 days.............4	
		After 28 days 5	

Qn No.	QUESTION	Fill or circle where applicable		GO TO Q.
318	Place of Death **Ekifo kyeyafiramu**			
	a) First birth	Gvt Hospital………………..1<br>Gvt Health Centre……………2<br>Mission Hospital…………3<br>Mission Health centre…………4<br>Private Hospital…………6<br>Private clinic…………7<br>Home…………………8<br>Along the way………..9<br>TBA…………………10	**Only applicable if 314 a) is 2**	
	**b) Second birth**	Gvt Hospital………………..1<br>Gvt Health Centre……………2<br>Mission Hospital…………3<br>Mission Health centre…………4<br>Private Hospital…………6<br>Private clinic…………7<br>Home…………………8<br>Along the way………..9<br>TBA…………………10	**Only applicable if 314 b) is 2**	

SECTION 4. PERSONAL EXPERIENCE RELATED TO LAST PREGNANCY

In the next three sets of questions, I am going to be asking about your experiences related to the three phases women go through when having a child that we discussed earlier: pregnancy, birth, and the period after birth. I'd like to begin by speaking with you about your last pregnancy that resulted in a baby (born alive or born dead). ***Mubibuzo ebyemirundi esatu egiraku, ndija kukubuza kubyobisemu ebigema kubintu ebyemirundi omukazi byabitamu nga azaala omwana gwetwogeireku: amabunda, okuzaala, nekisera nga omaze okuzaala. ndikwenda kutandikira kukwogera niiwe kubigema kumabundago agavaamu omwana (eyazalibwa nga mulamu oba nga mufu).***

Qn No.	QUESTION	Fill or circle where applicable	GO TO Q.
401	Did you visit health facility for antenatal care during the last pregnancy?<br>***Wakyalira eidwaliro okunwa obulezi nga oli mabundha agakasembayo?***	YES.................................1<br>NO 2	*If yes, go to Qn 403*
402	Why did you not see a health professional for antenatal care during your last pregnancy?<br>***Lwaki tiwabona musawo omukugu okunwa eidhagala kumabundha agakasembayo?***<br><br>**Probe Anything else**<br>**Circle all mentioned**	Did not know where to go…………...1<br>Health facility too far ....2<br>Too expensive.................................3<br>No one was there to accompany…….4<br>No good service...............................5<br>Others (specify)……………………..99	*Go to Qn 414*

Qn No.	QUESTION	Fill or circle where applicable		GO TO Q.
403	How many times in total did you receive antenatal care during your last pregnancy from the skilled health worker? **Wanwa obulezi mirundi emeka kumabundha agakasembayo?**	NO. OF TIMES……………………..		
		Don't know…………………………98		
404	How many months pregnant were you when you first received antenatal care during the pregnancy? ***Watandika okunwa obulezi nga amabunda gamyezi emeka nga?***	**[ ]/[ ]** Months		
		Don't know…………………………98		
405	Where did you attend most of your ANC from??	***Gha ghewasinga kunwera bulezi?***	**Facility Name (Ask the respondent the name of the health facility where she mainly attended ANC from**	
		Gvt Hospital………………..1		
		Gvt Health Centre……………2		
		Mission Hospital…………3		
		Mission Health centre…………4		
		Private Hospital…………5		
		Private clinics…………6		
		Drug shops ……………..7		
406	What is the distance from your home to where you attended Antenantal Care? ***Ghaligho buwanvuki okuva mumakago okutuka yewanwera obulezi?***	**[ ]/[ ]/[ ]** Miles		
407	What mode of transport did you use most while going for ANC? ***Ntambula kikaki yewasinga okukozesa nga oja okunwa obulezi?*** <br>**PROBE: What type of transportation did you mainly use to get to the Place of ANC?**	Ambulance……………............ 1		
		Private car 2		
		Taxi/bus 3		
		Boda-Boda Motorbike 4		
		Own Motocycle…………5		
		On foot ……………............... 6		
		Boda-Boda Bicycle 7		
		Own Bicycle………..8		
		Others (specify………………………..99		
408	Where did you get the money to pay for transport and other costs you incurred for ANC during your last pregnancy? **Wafunagha sente dewasasulira entambula nebintu ebindi byewasasulira nga oja okunwa obulezi kumabundha agasembayo?** <br>***Circle all mentioned***	Husband ……………………..…1		
		Personal saving (safety box, bank account)………………….…..2		
		Income generating activity/Business………..3		
		Sold an item at home……………..4		
		Got a loan/borrowed from saving group, ……………..5		
		Got a loan/borrowed from other sources………………..6		
		Savings from Saving group………7		
		Others (specify)……………………...99		

Qn No.	QUESTION	Fill or circle where applicable	GO TO Q.
**409**	As part of your antenatal care during the last pregnancy, was weight taken?<br>**Nga ekimu kubikolebwa nga ogire okunwa obulezi kumabunda agasembayo, wapimibwaku obuzito**	Yes ……….1 No……....2	
**410**	As part of your antenatal care during the last pregnancy, was blood pressure measured?<br>***Nga ekimu kubikolebwa nga ogire okunwa obulezi kumabunda agasembayo, wekeberwaku pulesa?***	Yes ……….1 No……....2	
**411**	As part of your antenatal care during the last pregnancy, was Urine sample taken?<br>**Nga ekimu kubikolebwa nga ogire okunwa obulezi bagakebera amaine?**	Yes ……….1 No……....2	
**412**	As part of your antenatal care during the last pregnancy , was blood sample taken?<br>**Nga ekimu kubikolebwa nga ogire okunwa obulezi kumabunda agasembayo, bakukebera omusaayi?**	Yes ……….1 No……....2	
**413**	As part of your antenatal care during the last pregnancy, was height taken?<br>**Nga ekimu kubikolebwa nga ogire okunwa obulezi kumabunda agasembayo, bakupima obuwanvu?**	Yes ……….1 No……....2	
**414**	During your last pregnancy, did you experience any of these problems?<br>***Nga oli mabunda agasembayo, wafunaku kubuzibu buno wansi?***<br><br>***Probe by reading the responses and circle all mentioned***	None……………………………….1	**If none, go to Qn 418**
		Bleeding…………….………………..2	
		Severe headache…………………3	
		Blurred vision ……………………..4	
		Convulsions…………..................5	
		Swollen hands/face/feet...………6	
		Fever …………...…………..….7	
		Unconsciousness.……..............8	
		Difficult breathing ……………...9	
		Severe weakness ……….….…..10	
		Severe abdominal pain………..11	
		Accelerated/ reduced Fetal movement…….…12	
		High blood pressure……………13	
		Water breaks Without labor ……….14	
		Others (specify)_________________99	
**415**	Did you seek assistance for any of the problem above?<br>***Wanonia obwidandabi kukizibu ebyo waigulu?***	Yes 1	***if no, go to question 417***
		No 2	

Qn No.	QUESTION	Fill or circle where applicable		GO TO Q.
416	If yes, Where did you seek assistance from? ***Bwoba nga wanonia obwidandabi, wabunonia gha?*** ***PROBE FOR THE TYPE OF FACILITY*** ***Circle all mentioned*** ***Make sure you probe to differetiate drug shops or clinics from the health centres***	Gvt Hospital…………1		
		Gvt Health Center..................2		
		Mission Hospital…………..3		
		Mission Health Centre…...............4		
		Private Hospital…………..5		
		Private Health Centre…...............6		
		TBA.....................................7		
		Community health worker 8		
		Relative/friend ……….............9		
		Drug shops/clinics ------------------10		
		Others (specify)_________________99		
417	Why did you not seek assistance for this problem? ***Lwaki tiwanonia bwidandabi kukizibu kino?*** ***PROBE FOR OTHER REASONS*** **Circle all mentioned**	Resp. didn't think necessary 1		
		Husband/family didn't think necessary 2		
		Facility too far 3		
		No transport 4		
		No childcare 5		
		Too expensive.......................... 6		
		Services are poor.......................7		
		Used home remedy....................8		
		Did not know where to go...............9		
		Had no time to go 10		
		Others (specify)________________99		
418	During the last pregnancy, did you use a mosquito net? ***Wakozesa akatimba k'ensiri ku mabundha agakesembayo?***	Yes……….1 No…………2		
419	Did you take any fansidar (drug) to prevent malaria? ***Wamira amakerenda ga fansida okwetangira omusuudha?***	Yes……….1 No…………2		
420	Were you given an injection (TT) in the arm to prevent the baby from getting tetanus during the last pregnancy? ***Bakukuba akapiso ka (TT) kumukono okutangira omwana obutafuna tettanus ku mabundha agakasembayo?***	Yes……….1 No…………2		
421	Did you take any drugs to prevent anemia (iron tablets or folic acid or syrup)? **Wamiraku amakerenda agaizamu omusaayi?**	Yes……….1 No…………2		

Qn No.	QUESTION	Fill or circle where applicable	GO TO Q.
422	For how long did you take any drugs that prevent anemia (iron tablets or folic acid or syrup)? **Watwala ibangaki nga omira amakerenda agaizamu omusaayi?**	Less than one month……………..1	
		One month and above…………2	
423	Before the time of delivery had you bought/ did you have the following; ***Nga wabulayo ewiki ndhala ozale, wali oguze / nga olinha binho wamanga;***		
	(a) Gloves ***Giravusi***	Yes………..1 No…………..2	
	(b) Mackintosh/kavera ***Ekiveera***	Yes………..1 No…………..2	
	(c) Razorblade ***Ekereta***	Yes………..1 No…………..2	
	(d) Thread for tying the cord ***Akawuuzi akasiba omudondo***	Yes………..1 No…………..2	
	(e) Tetracycline ***Akalezi akamaiso***	Yes………..1 No…………..2	
	(f) Syringes and needles ***Ekirawuli n'empiso***	Yes………..1 No…………..2	
	(g) Cotton or gauze ***Pamba***	Yes………..1 No…………..2	
424	Which items/materials did you buy for this pregnancy that you have not bought for the previous pregnancies that you have had? **Waliyo ebintu ebyokukozesa byewagula kumabunda gano nga tiwabigula kumabunda ageinuma?**		
	(a) Gloves ***Giravusi***	Yes………..1 No…………..2	
	(b) Mackintosh/kavera ***Ekiveera***	Yes………..1 No…………..2	
	(c) Razorblade ***Ekereta***	Yes………..1 No…………..2	
	(d) Thread for tying the cord ***Akawuuzi akasiba omudondo***	Yes………..1 No…………..2	
	(e) Tetracycline ***Akalezi akamaiso***	Yes………..1 No…………..2	
	(f) Syringes and needles ***Ekirawuli n'empiso***	Yes………..1 No…………..2	
	(g) Cotton or gauze ***Pamba***	Yes………..1 No…………..2	
425	During the last pregnancy, did you receive health education about the following? ***Kumabundha agakasembayo, wawuliraku kubinho wamanga?***		
	Danger signs during pregnancy **Obubonero obubi nga oli mabunda**	Yes………..1 No…………..2	
	How to feed yourself **engeri yoyinza okwerisamu**	Yes………..1 No…………..2	
	Preparing for birth **okwetegekera okuzaala**	Yes………..1 No…………..2	
	Attending ANC **Okuja okunwa obulezi**	Yes………..1 No…………..2	
	Delivering from the health Facility **Okuzaalira mwidwaliro**	Yes………..1 No…………..2	
426	Where did you get the information from? ***Amawulire wagatolawa?***	Community health worker ……………1	
		Radio…………………..2	
		Health worker/Health Facility 3	
		Friend/Relatives ………………4	
		Community meetings………………..5	
		Others (specify)………………………..99	

Qn No.	QUESTION	Fill or circle where applicable		GO TO Q.
427	Did the community health worker/VHT visit you at home while pregnant?<br>*Omusawo omuwi wamagezi kukyala yakukyaliraku mumakago nga oli mabunda?*	Yes..........1 No............2	*if no, go to section 5*	
428	How many times did the Community Health Worker visit you while pregnant at home?<br>**Mirundhi emeka abasawo abokukitundu gyebakukyariraku waka nga olimabundha?**	[ ]/[ ] Times		

SECTION 5. PERSONAL EXPERIENCE RELATED TO LAST BIRTH (During delivery)

Now I'd like to speak with you about the birth that resulted from the pregnancy we were just discussing ***Buti nsaba kwogeraku niiwe kuluzalo lwetuva okwogerangaku***

501	How did you give birth? **wazala otya?**	Ceasrian................1	
		Normal.................2	
		Assisted Using Instruments.........3	
502	Where did you give birth to your last child?<br><br>**wazalira gha omwanawo asembayo?**	Respondent's home................... 1	
		TBA's home 2	
		Other home 3	
		Gvt. hospital 4	
		Gvt. health center. 5	
		Pvt. hospital 6	
		Private health centre/clinic...............7	
		Mission hospital 8	
		Mission health centre...............9	
		On my way to facility............10	
		Drug shops...................11	
		Others (specify).........................99	
503	Who helped you deliver?<br><br>***Ani eyakuyamba okuzalisa?***	Doctor 1	
		Nurse/Midwife............. 2	
		Clinical Officer 3	
		Nursing Assistant............4	
		Community Health Workers/VHT........ 5	
		TBA 6	
		Relative or Friend........... 7	
		Delivered on my own................8	

Qn No.	QUESTION	Fill or circle where applicable	GO TO Q.
504	Can you tell me reasons why you did not give birth in a health facility?<br>**Osobola okunkoberaku kunsonga lwaki tiwazalira mwidwaliro?**<br><br>***PROBE: What else?***<br><br>**Circle all mentioned**	Respondent didn't think necessary .... 1<br>Husband/family didn't think necessary .... 2<br>Facility too far........................ 3<br>No transport means .... 4<br>No money for transport…………….5<br>No one to care for children .... 6<br>Facility services too expensive...................... 7<br>Services are poor........... 8<br>Did not know where to go... 9<br>Labor progressed too fast......... ……10<br>Other specify…………….99	
505	Did you receive any visit from the Community Health Worker/VHTs after deliverly<br>***Wafunaku okukyalirwa omusawo omuwi wamagezi kukyalo nga omaze okuzaala?***	Yes............................. 1<br>No 2	***If no, go to Qn 508***
506	If yes in 505, how many times did the Community Health Worker visit you at home after delivery?<br>***Bwoba yii mu 505, mirundi emeka omusawo omuwi wabagezi mukyalo gyeyakukyalira aghaka nga omaze okuzaala***	**[ ]/[ ]** Times	
507	After birth, when did the VHT first visit you?<br><br>***Omusawo omuwi wamagezi kukyalo yakukyaliraku nga ghabisewo ibangaki nga omaze okuzaala?***	Within 48 hours…………..1<br>After 48 hours…………2	
508	Prior to this birth, did you or your family make any arrangements for the birth of this child?<br>***Mukwetegekera okuzaala kuno, obomumakago bakola entegeka kukuzaala omwana ono?***	Yes............................. 1<br>No 2	***If no, go to question 510***
509	Which arrangements did you or your family make for the birth of this child?<br>**Ntegekaki abomumakago yebakola okutegekera okuzaala omwana ono?**<br><br>***PROBE: What else?***<br><br>**Circle all mentioned**	Identified Transport………...1<br>Individual Saving……………..2<br>Saved Money with a Saving group……3<br>Bought Birth Items ………….4<br>None……………………......5<br>Others (specify)………………………..99	
510	What is the distance from your home to where you delivered?<br>***Buwanvu ki obuliwo okuva wakawo okutuka gyewazalira?***	**[ ]/[ ]/[ ]** Miles	

Qn No.	QUESTION	Fill or circle where applicable	GO TO Q.
511	What was the mode of transport used to the place were you delivered from? **Wakozesa ntambulaki okuja mukifo yewazalira?**	Ambulance............................... 1	
		Private car 2	
		Taxi/bus 3	
		Boda-Boda Motorbike 4	
		Own Motocycle...............5	
		On foot 6	
		Boda-Boda Bicycle 7	
		Own Bicycle................8	
		Others (specify)............................99	
512	Did you pay any money for the transport **Wasasula sente yona yona kuntambula?**	Yes............................. 1	**If no, go to Qn 514**
		No........................2	
513	What was the main source of money you used to pay for transport to facility? ***Watolagha sente dewasasula kuntambula okuja mwidwaliro?***	Husband paid.............................1	
		Personal saving...........................2	
		Income generating activity.......3	
		Sold an item at home......................4	
		Got a loan/borrowed......................5	
		Funds from the saving group..............6	
		Others (specify)............................99	
514	Did you pay any money to those who assisted you with delivery (health unit or health workers / TBA)? **Wasasula sente yona yona eri abo abakuyamba okuzalisa(kwirwaliro oba omusawo/mulerwa)**	Yes............................. 1	**If no, go to Qn 517**
		No 2	
515	How much did you pay? **Wasasula imeka?**	_________________Ush	
516	What was the main source of money you used to pay the person who assisted you to the health facility and other costs you incurred ? **Watolagha sente dhewakozesa okusasula okusasula omuntu eyakuyamba okuja mwidwaliro nebindi byewasansaniaku?**	Husband paid.............................1	
		Personal saving...........................2	
		Income generating activity..................3	
		Sold an item at home......................4	
		Got a loan/borrowed......................5	
		Funds from the saving group..............6	
		Others (specify)..........................99	
	Now, I would want us talk about the newborn care practices that were done at the place where you delivered from.		
517(a)	Did you put anything on the baby's cord ***Wataku ekintu kyona kyona kumudondo?***	Yes............................. 1	***If no, Go to Qn 518***
		No...................... 2	

Qn No.	QUESTION	Fill or circle where applicable	GO TO Q.
517(b)	What did you put on the cord to ensure that it dired well or fast?<br>**Kiki kye wata kumudondo okuwonha nga amaze okuzalibwa?**<br>***PROBE: What else?***<br>**Circle all mentioned**	Washed with soap and put nothing…1<br>Medical drugs……………………..2<br>Powder…………………………3<br>Ash………………………………4<br>Salty Water………………………..5<br>Surgical spirit……………………6<br>Others (specify)……………………99	
518	Was the baby weighed immediately after birth?(ask for the Card to check)<br>**Omwana bamupimawo nga bakamuzala?**	Yes……………………..1<br>No……………………..2<br>Don't know………………………..98	***If no or don't know, go to 520***
519	How much did (Name) weigh? Record weight from health card, if available. .<br>**(Erina) yavamu kilo imeka?**	………………..Kgs	
520	Do you think or were you told that the baby was very small? ***Olowooza oba wakoberwaku nti omwana yali mutono ino?***	Yes………..1<br>No…………2	***If, no go to Section 6***
521	If the baby was small; Did you make extra visits to the health facility because your baby was small?<br>**Wagyaku mwiidwaliro emirundhi mingi olw'okuba omwana yalimutono?**	Yes……………………..1<br>No……………………..2	
522	If the baby was small; Were you given any information on how to care for small babies by CHW/person who assisted in delivery?<br>***Olwokuba omwana yali mutono, abasawo abokukitundu / oyo eyakuyamba mukuzalisa yakukoberaku engeri gyebalabiliramu omwana omutono?***	Yes……………………..1<br>No……………………..2	***If no, go to Qn 524***
523	*What were you told to do?*<br>***Bakukobera ki?***<br>***PROBE: What else?***<br>***Circle all mentioned***	More frequent breastfeeding……………1<br>Skin-to-skin care………………..2<br>Delay first bath for a week or longer………3<br>Take baby to health facility………………4<br>Extra PNC visits……………5<br>Others (specify)…………………………99	
524	Because your baby was small, was [NAME] taken to a health facility for special care?<br>**Olwokuba (Name) yalimutono ,yatwalibwaku mwidwaliro okufuna endhabilira eyendhawulo?**	Yes……………………..1<br>No………………………..2	**If no, go to section 6**
525	Did (NAME) receive Kangaroo Mother Care (KMC) at a health facility (Probe: by KMC I mean……….)<br>***(Erina) yatebwaku mukifuba kyo nga alimwidwaliro?***	Yes……………………..1<br>No………………………..2	**If no, go to section 6**

Qn No.	QUESTION	Fill or circle where applicable	GO TO Q.
526	For how long did they do the kangaroo care?<br>**Endabirira eyokughemba omwana mukifuba bagikolera ibangaki?**	Days____________<br>Weeks_____________	

SECTION 6. PERSONAL EXPERIENCE RELATED TO LAST BIRTH (POSTPARTUM)

Now I'd like to speak with you about the period after the birth we were just discussing.
Ndikwenda kukubuuza kubigema kukiseera nga omaze okuzaala kwetwakamala okwogeraku.

Qn No.	QUESTION	Fill or circle where applicable	GO TO Q.
601	Was the baby put skin to skin immediately after birth?<br>***Nga omaze okuzaala omwana wamutakuku lususu kulususu?***	Yes……………………..1<br>No………………………..2	**If No, go to Qn 603**
602	For how long was this done?<br>***Kino kyakolebwa ibangaki?***	_____________Minutes<br>__________Hours	
603	When did you first bathe the baby after delivery?<br>**Omwana wasoka omunaza nga wabise ibangha ki nga amaze okuzalibwa?**	Immediately (less than 1hour)…………1<br>Within 1- 6 Hours……………………….2<br>Between 7 -24 Hours…………………3<br>After 24 Hours …………………………4	
604	When did someone first check on your health after you gave birth?<br>**Bakukeberaku nga wabise ibanghaki nga omaze okuzala?**	0- 6 Hours.............. 1<br>7 hours- 6 days................. 2<br>7 days- 6 weeks .... 3<br>None……………………4	***If 4, go to Qn 606***
605	Who checked on your health at that time?<br>**Ani eyakukeberaku ekiseera ekyo?**<br>***Circle all mentioned***	Doctor .... 1<br>Nurse/Midwife.............. 2<br>Clinical Officer .... 3<br>Community Health Workers........... 4<br>TBA .... 5<br>Relative or Friend............ 6<br>None……………………7	

Qn No.	QUESTION	Fill or circle where applicable	GO TO Q.
606	Immediately after delivery was the following done:<br>**Nga omazze okuzala,bakolaku binho wansi:**<br>***PROBE: What else?***		
	Vitamin A supplementation to the mother **Akakerenda akawebwa omukazi nga amaze okuzaala okuyamba omwana okafuna mumabere**	Yes……………1 No…………………2	
	Immunization for the baby **Okugema omwana**	Yes……………1 No…………………2	
	Others (specify)______________________	Yes……………1 No…………………2	
607	How long after birth did you FIRST put the baby to the breast?<br>**Omwana wasoka okumwonsa nga wabise ibanghaki nga omazze okuzala?**	Immediately (less than 1hour)…………1	
		Within 1- 6 Hours………..2	
		Within 7 – 24 Hours……..3	
		After 24 Hours ……….4	
608	Did you give anything else in the first month apart from breast milk to feed the baby?<br>***Omwana olinayo ekintu ekindhi kyewamuwa nga otoileku amabere mumwezi omulala ogwasoka?***	Yes……………………………1	***If no, go to Qn 610***
		No………………………………2	
609	What was given apart from breast milk to feed the baby?<br>***Kiki ekindhi kyewamuwa ekitali mabere?***<br>***PROBE: Anything else? Circle all responses given***	Milk (other than breast milk)…………1	
		Plain water……………………..…2	
		Sugar or Glucose water………4	
		Salt solution/soup………..…5	
		Fruit juice…………………..……6	
		Tea……………………..…….7	
		Honey……………………..…8	
		None………………..9	
		Others (specify)……………………………..99	
610	Did you go back to the hospital/health centre within the first 6 weeks after you had the baby for a medical check up on your health or your baby's health?<br>***Wairayo mwidwaliro oluvainhuma olwesabiiti 6 nga omaze okufuna omwana omusawo okukebera kubulamubwo oba obw'omwanawo?***	Yes……………………………1	***If no, go to Qn 618***
		No………………………………2	
611	If yes, how many times did you visit the health facility after delivery?<br>***Bwoba wairayo, wairayo emirundi emeka mwidwaliro nga omaze okuzaala***	___________Number of times	
612	What were the reasons? **Nsongaki edhakwizayo?**<br><br>**Circle all mentioned**	Immunizations ……..………1<br>Baby was sick……………………..2<br>Mother was sick…………………………3<br>Review of the baby and Mother…………4	
		Others (specify)_________________99	

Qn No.	QUESTION	Fill or circle where applicable		GO TO Q.
613	Whose health was checked at this visit (Within 6 weeks after delivery)?<br>***Bulamu bwani obwakeberwa kumulundi guno?***	Baby………………..1<br>Mother……………2<br>Both baby and the mother……………..3		
614	How did you travel to the hospital (While going for first PNC after delivery)?<br>***Watambula otya okutuuka kwidwaliro (nga oja okwirayo okukeberaku okwasooka)?***	Ambulance………………........... 1<br>Private car .... 2<br>Taxi/bus .... 3<br>Boda-Boda Motorbike .... 4<br>Own Motocycle……………5<br>On foot ……….......................... 6<br>Boda-Boda Bicycle .... 7<br>Own Bicycle……………..8<br>Others (specify)…………………...99		
615	Did you pay any money for the transport for the first PNC visit after delivery?<br>***Wasasula sente edentambula kumulundi ogwokubiri gwewaja?***	Yes……………………1<br>No……………………..2		**If no, go to Qn 618**
616	How much did you pay (Ug sh**)** for travelling to the health facility? ***Wasasula sente imeka kuntambula okuja mwidwaliro?***	…………………………...Ush		
617	What was the main source of money you used to pay for transport to facility?<br>***Watolagha sente dewasasula kuntambula okuja mwidwaliro?***	Husband paid……….…………….1<br>Personal saving…………….……..2<br>Income generating activity……………….3<br>Sold an item at home…………………..4<br>Got a loan/borrowed…………………..5<br>Funds from the saving group…………..6<br>Others (specify)………………………..99		
618	Why didn't you visit a health facility after delivery within the first 6 weeks?<br>***Lwaki wakyalira eidwaliro nga omaze okuzaala?***<br><br>***This should be asked to only women who never went for PNC***<br><br>***PROBE: What else?***<br><br>**Circle all mentioned**	I didn't think it was necessary…. .... 1<br>Husband/familly didn't think it was necessary .... 2<br>Facility too far .... 3<br>No transport .... 4<br>No one to care of Children. .... 5<br>Facility services too expensive..................... 6<br>Services are poor .... 7<br>Used Home Remedy......... 8<br>Did not know where to go........ 9		

Qn No.	QUESTION	Fill or circle where applicable		GO TO Q.
		No time to go 10		
		Bought drugs from drug shop…………..11		
		Others (specify)…………………………..99		
	**Now I would like us talk about the danger signs you experienced after delivery**			
**619**	Did you experience any danger sign after birth? **Wafunaku obubonero obubi nga omaze okuzaala?**	Yes……………………………1		***If no, go to Qn 621***
		No………………………………2		
**620**	If yes, which danger signs? ***Bwobanga wafuna obubonero obubi, buboneroki?***	Severe Bleeding ………1		
		Severe Headache…….2		
		Blurred Vision………..3		
	***PROBE: What else? ***	Convulsions……….4		
		Swollen Hand/Faces/body/feet……..5		
	**Circle all mentioned**	Fever……..6		
		Bad smell Vaginal Discharge……7		
		Loss of Consciousness……..8		
		Difficulty Breathing………9		
		Severe weakness……..10		
		None………………………11		
		Others (specify)…………………………..99		
**621**	Did you go to the health facility for any of the above problem(s)? ***Waja mwidwaliro kubuzibu obwo ghaigulu?***	Yes.................... 1		***If yes, go to Qn 623***
		No...................... 2		
**622**	Why did you not seek assistance for the above danger sign(s)? ***Lwaki tiwanonia buyambi kububonero obwo ghaigulu?*** ***PROBE: What else?***	I didn't think it was necessary…. 1		
		Husband/familly didn't think it was necessary 2		
		Facility too far 3		
	**Circle all mentioned**	No transport 4		
		No one to care of Children. 5		
		Facility services too expensive...................... 6		
		Services are poor 7		
		Used Home Remedy......... 8		
		Did not know where to go........ 9		
		No time to go 10		
		Bought drugs from drug shop…………..11		
		Others (specify)…………………………..99		

Qn No.	QUESTION	Fill or circle where applicable	GO TO Q.
623	Did the baby have any danger sign?<br><br>*Omwana omuwere yafunaku obubonero obubi?*	Yes.................... 1<br>No...................... 2	*If no, go to Qn 627*
624	Which danger signs did your baby experienced ?<br>*Buboneroki obubi omwana bweyafuna?*<br><br>***Probe: Anything else?***<br><br>**Circle all mentioned**	Difficult or Fast breathing ..... 1<br>Yellow skin/ Eye color (Jaundice)......... 2<br>Poor sucking or feeding .... 3<br>Pus, Bleeding or Discharge from around the Umbilical cord .... 4<br>Baby Very Small............................ 5<br>Skin Lesions or Blisters .... 6<br>Convulsions ...... 7<br>Spasms /rigidity/stiff……………..8<br>Lethargy/Unconsciousness........ 8<br>Red/Swollen Eyes with Pus ... 9<br>Others (specify)…………………………..99	
625	Did you seek assistance for the above danger sign(s)?<br>*Wanonia obuyambi kubuzibu obwo ghaigulu?*	Yes.......................... 1<br>No............................ 2<br>Don't know...................... 98	*If yes, go to Qn 627*
626	Why did you not seek assistance for the above problem(s)?<br>*Lwaki ti wanonia buyambi kubuzibu obwo ghaigulu?*<br><br>***PROBE:***<br><br>**Circle all mentioned**	I didn't think it was necessary…. .... 1<br>Husband/familly didn't think it was necessary .... 2<br>Facility too far ……............ 3<br>No transport ……............... 4<br>No one to care of Children. .... 5<br>Facility services too expensive...................... 6<br>Services are poor .... 7<br>Used Home Remedy......... 8<br>Did not know where to go…..... 9<br>No time to go .... 10<br>Bought drugs from drug shop…………..11<br>Others (specify)……………………………..99	

Qn No.	QUESTION	Fill or circle where applicable		GO TO Q.
627	Approximately how long did you take before resuming your regular work after you delivered?(if less than a day code hours, if less than a week code days, if less than a month code weeks else code months) ***Okugerageraniawatwala ibangaki nga okali kwiramu kukola mirimojo ejabulidho nga omaze okuzaal, (bwekiba tikigheza lunaku laga sawa, bwekiba tikigheza wiiki laga naku, bwekiba tikigheza mwezi laga wikis ebindi laga myezi)***	[ ]/[ ] Months<br>[ ]/[ ] Weeks<br>[ ]/[ ] Days<br>[ ]/[ ] Hours		
628	If you are in employment or participate in other income generating activities (eg selling vegetables, working in the fields) how much time did you take away from your work while you were away to give birth, and while recovering? ***Bwobanga olinomulimo oba nga wetaba mubintu ebindi ebingiza sente (okugeza nga okutunda ebibala, okukola munimiro,) ibanga ki lyewamala ngatoli kumulimogwo nga ogiire mukuzaalanga okaali kuwon?***	[ ]/[ ] Months<br>[ ]/[ ] Weeks<br>[ ]/[ ] Days		
629	What would you normally have been doing during the time you were in labor/delivery and during the time you were recovering from delivery? ***Walibaire okalaki mubisera byewabereramu nga olikulumw/okuzaala nekisera kyewabereramu nga olikuwona nga omaze okuzaala?***	Household chores . . . . 1<br>Working on the household farm. .. .2<br>Petty trading . . . . 3<br>Employed as a skilled Labourer . . . 4<br>Employed as an unskilled Labourer . . 5<br>Clerical/Professional work . . . . .6<br>Other specify 99		
630	If you are in employment or participate in other income generating activities (eg selling vegetables, working in the fields) how much time did you take away from your work while you were away to give birth, and while recovering? ***Bwoba nga okozesebwa oba nga wetaba mukintu kyona kyona ekingiza sente (okugeza nga okutunda ebibala, okukola mumisiri) wamala ibangaki nga toli kumulimogwo nga ojire okuzaala nekiseera kyewamala okuwona?***	[ ]/[ ] Months<br>[ ]/[ ] Weeks<br>[ ]/[ ] Days<br>Maternity leave enter 00 [ ]/[ ]		
631	*How much would you have earned from your employment or income generating activities during the time you were away from work to give birth, and during recovery?* **Wandibaire ongiza sente imeka kumulimogwo oba mubintubyo ebireta sente kukisera kyewamala nga ozirawo nga ojire okuzaala nikyewatwala okuwona?**	Ushs_____________Daily<br>Ushs___________Weekly<br>Ushs____________Monthly		

Qn No.	QUESTION	Fill or circle where applicable		GO TO Q.
632	In order of importance, list from the options below the 3 activities you normally would have been doing while you were away to give birth and during recovery. ***Mumitendera egyemigaso, laga ebintu ebisatu ebisinga kwebyo byotera okukola kyewalibaire okola mukisera kyewabereramu nga ogiire okuzaalanikyewatwaala okuwona .*** **Options for activities** Household chores . . . . 1 Petty trading . . . . 2 Skilled labour . . . . 3 Unskilled labour . . . . .4 Professional work. . . . . .5 Other specify 99	1 [ ] 2 [ ] 3 [ ]		
633	Who took over this/these activity(ies)? (multiple responses possible circle all that apply) ***Ani eyatwala obuvunanizibwa kumirimogyo nga ozirawo?***	No one . . . . .1 Household members . . . . 2 Relatives . . . . 3 Friends/Neighbours . . . . 4 Hired Persons . . . . 5 Other Specify 99		**If no one, Go to section 7**
634	Did the person who took over this activity receive any payment in cash or kind? ***Omuntu eyatwala obuvunanizibwa buno yasasulwa mungeri yona yona oba yasente oba tiyasente?***	Yes . . . . .1 No . . . .2 Don't know 98		**If no & Don't know, Go to section 7**
635	How much payment did they receive (if payment is in kind, ask for approximate value) ***Basasulwa sente imeka (bwebabanga tibasasulwa sente, buza omuwendo okugeragerania mwekyo kyebasasula)***	_______________Ushs		

Section 7: FAMILY PLANNING

Qn No.	QUESTION	Fill or circle where applicable	GO TO Q.
701	Have you ever used any method to delay or avoid getting pregnant?. ***Wali okozeisaku enkola yonayona okulwawo okutola enda oba okwetangira okufuna amabunda?***	Yes……………………1 No……………………..2	*If no, go to Qn 705*
702	Which method have you ever used?(PROBE for each method, select all mentioned) ***Nkolaki yewali okozeisaku?***	Female sterilization…………………..1 Male sterilization………………2 IUD………………………3 Injectables………………4 Implants……………5 Pill…………………6 Male condom…………………7 Female condom…………………..8 Diaphragm…………………..9 Foam/Jelly……………..10	

Qn No.	QUESTION	Fill or circle where applicable	GO TO Q.
		Lactational Amenorrhea Method……………11	
		Rhythm method…………12	
		Withdrawal…………..13	
		Other method (specify)……………………..99	
**703**	Are you currently using any method to delay or avoid getting pregnant?. ***Olinenkola yona yona elwisawo oba okutangira okufuna enda?***	Yes……………………1	***If no, go to Qn 705***
		No……………………..2	
**704**	Which method are you currently using?(PROBE for each method, select all mentioned) ***Nkolaki yokozesa buti?***	Female sterilization…………………..1	
		Male sterilization………………2	
		IUD………………………3	
		Injectables………………4	
		Implants……………5	
		Pill…………………6	
		Male condom…………………7	
		Female condom…………………..8	
		Diaphragm…………………..9	
		Foam/Jelly……………..10	
		Lactational Amenorrhea Method……………11	
		Rhythm method…………12	
		Withdrawal…………..13	
		Other method (specify) 99	
**705**	Why are you not using any methord to delay or avoid getting pregnant? ***Lwaki tokozesa famire?*** ***PROBE: What else? Circle all responses given***	I didn't think it was necessary…. 1	
		Husband/familly didn't think it was necessary 2	
		I do not know where to access the services from 3	
		I am using traditional methods ….............. 4	
		I hear it has side effects 5	
		Our religion does not allow..................... 6	
		Have not yet seen my periods……….7	
		Others (specify)………………………..99	

Qn No.	QUESTION	Fill or circle where applicable	GO TO Q.
	**Section 8: SOCIO-ECONOMIC ISSUES**		
**801**	Does your household have the following items?<br>**Amaka gaimwe galinha binho waigulu?**		
	(a) Electricity/solar ***Amasanalaze***	Yes…………..1 No…………….2	
	(b) A radio ***Ladiyo***	Yes…………..1 No…………….2	
	(c) A cassette player ***Kaseti***	Yes…………..1 No…………….2	
	(d) A television ***tolofaina***	Yes…………..1 No…………….2	
	(e) A mobile phone ***eisimu eyomungalo***	Yes…………..1 No…………….2	
	(f) A fixed phone ***Eisimu eyokumeza***	Yes…………..1 No…………….2	
	(g) A refrigerator ***Filigi***	Yes…………..1 No…………….2	
	(h) A table ***Emeza***	Yes…………..1 No…………….2	
	(i) A chair ***Entebe***	Yes…………..1 No…………….2	
	(j) A sofa set ***Entebe eyekyoya***	Yes…………..1 No…………….2	
	(k) A bed ***Ekitanda***	Yes…………..1 No…………….2	
	(l) A cupboard ***Ekabada***	Yes…………..1 No…………….2	
	(m) Clock ***Esawa eyokukisenge***	Yes…………..1 No…………….2	
**802**	What type of fuel does your household mainly use for cooking?<br>**Amaka gaimwe gasinga kufumbisaki?**	Electricity / Solar……………………1	
		Lpg/natural gas…………………..2	
		Biogas …………………..3	
		Kerosene/paraffin……………………..4	
		Charcoal………………………….5	
		Firewood……………………….6	
		Straw/shrubs/grass………………..7	
		Animal dung………………………….8	
		No food cooked in household……..9	
		Other (specify) …………99	
**803**	What is the main source of lighting for the household?<br>***Amakago gasinga kwakisa ki?***<br><br>***Do not read answers. Circle only one***	Electricity………………………….1	
		Gas……………………….2	
		Lantern…………………..3	
		Tadoba…………………4	
		Solar………………..5	
		Candles………………6	
		Cow dung or grass reeds………….7	
		Firewood…………………8	

Qn No.	QUESTION	Fill or circle where applicable		GO TO Q.
		Dry cell torches…………...9		
		Others (specify)_______________99		
804	MAIN MATERIAL OF THE FLOOR ***(RECORD OBSERVATION)***<br>***Ebyasinga okukozesebwa okutandaaza munumba wansi***	**Natural floor**		
		Earth/sand…………………..1		
		Earth and dung…………….2		
		**Finished floor**		
		Parquet or polished wood……………..3		
		Mosaic or tiles……….4		
		Bricks…………..5		
		Cement……………6		
		Stones…………..7		
		Other (specify)……………….99		
805	MAIN MATERIAL OF THE ROOF (RECORD OBSERVATION)<br>***Ebyasinga kukozesebwa mukusereka***	Thatched (grass/fibre)………………………8		
		Wood/planks……………9		
		Iron sheets…………………10		
		Asbestos…………………11		
		Tiles……………….12		
		Tin…………….13		
		Cement………………14		
806	MAIN MATERIAL OF THE EXTERIOR WALLS ***(RECORD OBSERVATION)***<br>***Ebyasinga okukozesebwa okuzimba ebisenge kuluya***	Thatched/straw……………………1		
		Mud and poles…………..2		
		Un-burnt bricks with mud………………3		
		Un-burnt bricks with cement……………4		
		Burnt bricks with mud…………5		
		Burnt bricks with cement…………..6		
		Cement blocks…………..7		
		Stone……………..8		
		Timber…………..9		
		Other (specify)………………………..99		
806	What type of animals do you own? If yes, how many does the respondent's household own?<br>***Bisolo kikaki amaka gano byegali nabyo? Bwekiba nga yii, alikubuzibwa alinabimeka?***		***Number***	
	Goats ***Embuzi***	Yes……….1 No…………2		
	Cows ***Ente***	Yes……….1 No…………2		
	Sheep ***Entaama***	Yes……….1 No…………2		

Qn No.	QUESTION	Fill or circle where applicable	GO TO Q.
	Chicken/other birds (e.g. ducks) **Enkoko**	Yes……….1 No…………2	
	Turkeys **Sekoko**	Yes……….1 No…………2	
	Ox plough **Ente erima**	Yes……….1 No…………2	
	Pigs	Yes……….1 No…………2	
**807**	Do you own land? ***Olineitaka?***	Yes……….1 No…………2	***If no, go to Qn 809***
**808**	What is the size of the land? ***Eitaka lyagagha obunene?***	Plot…………………1	
		Half an acre…………..2	
		One acre………………..3	
		Above one acre…………4	
**809**	Does any member of this household own? **Waliwo omuntu yenha yenha mumaka gaimwe alinha**		
	(a) Bicycle ***Egali***	Yes……….1 No…………2	
	(b) Motorcycle/scooter ***Epiki piki***	Yes……….1 No…………2	
	(c) Animal drawn cart ***Ekigali***	Yes……….1 No…………2	
	(d) Car/Truck ***Emotoka/lore***	Yes……….1 No…………2	
	) A boat with a motor ***Elyato lya yingeni***	Yes……….1 No…………2	
	(f) A boat without a motor ***Elyato elitali lya yingeni***	Yes……….1 No…………2	

Thanks very much for your time. Now I would like to talk to your Husband and ask him/her some few question related to Maternal Health. I will not Share with him/her what we have discussed together. Could you please take me to you Husband

Webale inho olwobwire bwotughaire.Buti nhenda kwogeraku n'omwamiwa/akulabirira imubuuzeku ebibuuzo bitontono ebigema bulamu bwababakyala abazaire. tiidha kumukobera byetwogeire niiwe, osobola okuntwala yomwamiwo.

1. Husband Present Yes ( ) No ( ) ***Omwami/alabirira aliwo yiii( ) Bbee ( )***

2. When will he/she be around (if not present):___________ ***Anabaawoli***___________

3. Husband accepts to be interviewed Yes ( ) No ( ) ***Omwami/Alabirira aikiriza okubuzibwa Yii ( ) Bbee ( )***
